# Recyclable cooperative catalyst for accelerated hydroaminomethylation of hindered amines in a continuous segmented flow reactor

**DOI:** 10.1038/s41467-022-30175-0

**Published:** 2022-05-04

**Authors:** Malek Y. S. Ibrahim, Milad Abolhasani

**Affiliations:** Department of Chemical and Biomolecular Engineering, North Carolina State University, Raleigh, NC 27695 USA

**Keywords:** Homogeneous catalysis, Chemical engineering

## Abstract

Synthesis of hindered amines using the atom-efficient hydroaminomethylation (HAM) route remains a challenge. Here, we report a general and accelerated HAM in segmented flow, achieved via a cooperative effect between rhodium (Rh)/N-Xantphos and a co-catalyst (2-Fluoro-4-methylbenzoic acid) to increase the reactivity by 70 fold when compared to Rh/Xantphos in batch reactors. The cooperation between Rh and the co-catalyst facilitates the cleavage of the H–H bond and drives the equilibrium-limited condensation step forward. Online reaction optimization expands the scope to include alkyl, aryl, and primary amines. In-flow solvent tuning enables selectivity switching from amine to enamine without the need for changing the ligand. Furthermore, leveraging the ionic nature of the catalyst, we present a robust Rh recovery strategy up to 4 recycles without loss of activity.

## Abstract (web summary)

Flow chemistry enables intensified production of hindered amines. Here the authors present a rapid and reusable catalyst to operate in a segmented flow reactor for olefin hydroaminomethylation to selectively produce hindered amines or enamines.

## Introduction

Amines are essential building blocks in agrochemicals, detergents, and fine chemicals^1–3^. For example, alkylated anilines and carbazoles are used as chromophores in synthesis of organic light emitting diodes (OLEDs)^4,5^, alkylated phenylenediamines are applied as antioxidants in rubber, and long chain fatty amines are components in personal care and lubrication formulations. Moreover, the amine moiety is common in several drugs, including Ibutilide, Melperone, Terfenadine, Aripiprazole, Fluspirilene, and Difenidol^1^. Carbon–nitrogen (C–N) bonds are widely common in bioactive molecules and thus, rapid C–N bond formation reactions are essential tools for the generation of large molecular libraries for structure-activity-relationship mapping (SAR), and the on-demand synthesis of isotopically labeled tracers with relatively short half-life such as the ^11^C labeled PET tracers^6–8^. The emerging trend towards decentralized manufacturing of pharmaceuticals and fine chemicals promotes continuous flow production as the preferred method of choice^9–11^. Four conventional processes are typically used in the large scale synthesis of aliphatic amines: (1) amination of alcohols, (2) amination of alkyl halides, (3) reduction of nitrile compounds, and (4) reductive amination of carbonyl compounds^3^, which all require multistep processing and purification. HydroAminoMethylation (HAM) is an alternative one-pot synthetic route for sustainable manufacturing of amines from economic starting materials (olefins) without the need for the multistep rection-separation as needed in routes (1), (3), and (4) or the generation of metal halide solid waste that requires disposal as in route (2). HAM is an atom-efficient route as it produces no waste other than water as the sole byproduct.

**Fig. 1:**
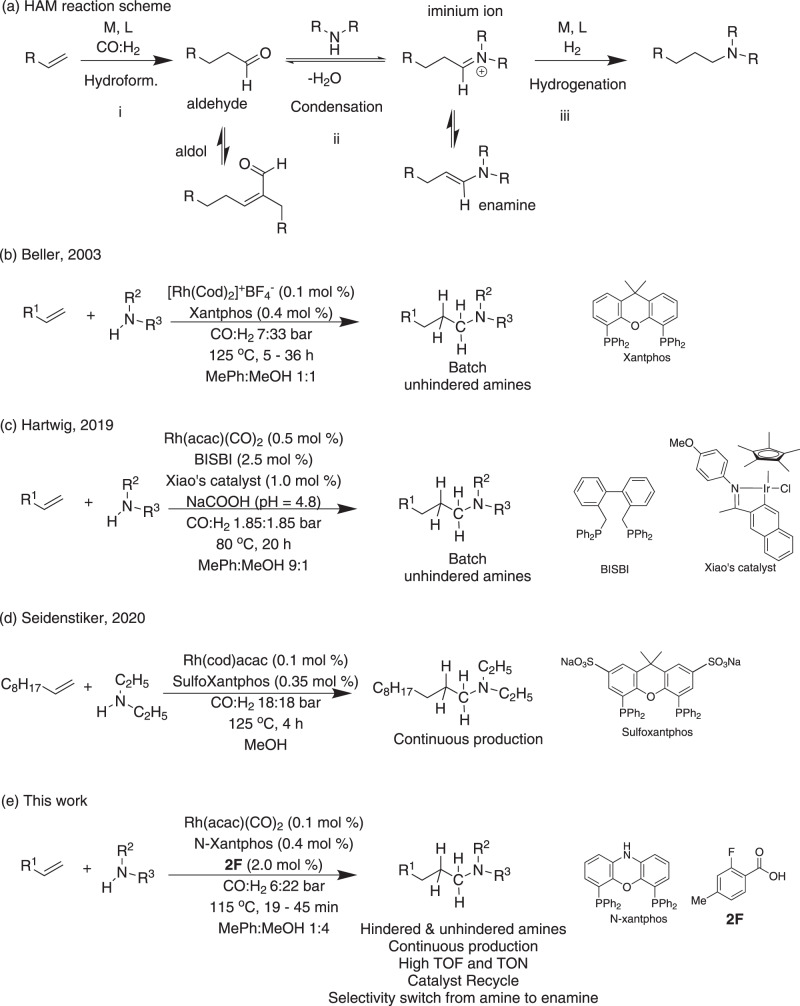
HAM reaction scheme and a summary of prior HAM work vs. this work. **a** Amine formation by tandem hydroformylation-condensation-hydrogenation reactions. M: metal, L: ligand. **b**–**e** Different approaches to amine syntheses via HAM reaction.

Substitution on the amine α carbon is often introduced in active pharmaceutical ingredients to control their lipophilicity and rate of metabolism^12^. However, hindered amines are usually synthesized by less economic routes such as the amination of alkyl halides. The HAM reaction proceeds through three main steps in an auto-tandem fashion (Fig. 1a): (i) hydroformylation, (ii) condensation, and (iii) enamine hydrogenation. Steps (ii) and (iii) are often referred to as the reductive amination of aldehydes. Steps (i) and (iii) are gas–liquid reactions and thus provide an exciting opportunity for intensification through flow chemistry (segmented gas–liquid flow format). Step (ii) is a liquid phase condensation reaction that can be equilibrium-limited with sterically hindered amines and thus the undesired aldehyde self-condensation becomes the major reaction. This is a significant drawback that has limited the scope of HAM reactions to non-hindered amines^13,14^. Recent studies have demonstrated advances in the rate acceleration and substrate scope expansion of HAM reactions^15,16^. However, examples of HAM with sterically hindered amines are rare, and alternative complex routes are developed specifically for their alkylation^17^. The cost associated with catalyst is another drawback that limits the use of HAM in large scale production. In addition to the increasing demand for expanding the substrate scope of HAM reactions to sterically hindered amines, there is a need for continuous operation of HAM with recycling the expensive rhodium (Rh) catalyst to enhance the process sustainability and promote decentralized manufacturing of pharmaceuticals and fine chemicals.

The application of the bidentate, commercially available phosphine ligand, Xantphos, in the Rh-catalyzed HAM reactions to maintain high selectivity towards the linear amine was pioneered by Beller and co-workers^18^. However, the HAM reaction is still plagued by the use of expensive cationic Rh(cod)^+^BF_4_^−^ catalyst and the slow kinetics (batch reaction time > 24 h at 115 °C, TOF < 200 mol/mol Rh/h) which makes it unsuitable for continuous synthesis (Fig. 1b). The reaction kinetics were enhanced by Hartwig and co-workers. through the addition of the iridium (Ir)-based Xiao’s hydrogenation catalyst and sodium formate to accelerate the enamine hydrogenation step (Fig. 1a, step iii), resulting in a complete conversion in 20 h at 80 °C (Fig. 1c)^19^. Despite the addition of 1 mol% of Xiao’s Ir catalyst, the hydroformylation step (Fig. 1a, step i) remained slow. To overcome the slow rate of the hydroformylation step, the Rh catalyst loading was increased by 5 times relative to the Rh loading in the study by Beller and co-workers, which resulted in TOF values below 50 mol/mol Rh/h. A more economic Rh source, Rh(acac)(CO)_2_, was used by Hartwig and co-workers. and the bidentate phosphene ligand, BISBI, showed better yields when compared to Xantphos under similar reaction conditions. Despite the significant advancements of the HAM synthetic route in batch reactions, no α-branched amines have been demonstrated. This major limitation arises from the equilibrium-limited condensation and slow hydrogenation of the branched enamine in batch reactors. Wasserscheid and co-workers demonstrated that Rh/Xantphos catalyst for continuous gas phase HAM reaction (TOF values up to 500 mol/mol Rh/h) could be recycled by immobilizing the catalyst in supported ionic liquid phase on activated carbon^20^. Recently, continuous HAM of 1-decene with the non-hindered diethylamine was demonstrated by Seidensticker and co-workers with catalyst recycling (0.1 mol% Rh loading and SulfoXantphos ligand) in a pressurized continuously stirred tank reactor (Fig. 1d)^21^. TOF values up to 200 mol/mol Rh/h was achieved and the catalyst recycle was accomplished by carrying out the reaction in a thermomorphic multiphase solvent system which allowed for product/catalyst separation upon reaction cooling.

In this work, we present a synthetic route for accelerated HAM reactions with hindered amines by leveraging a cooperative effect between Rh/N-Xantphos and fluorinated benzoic acid catalyst. Compared to the traditional Rh/Xantphos system, the developed cooperative catalyst increases the kinetics of HAM reactions by up to 70 fold when conducted in flow, while achieving high linear/branched selectivity (*l*/*b*). Furthermore, we demonstrate HAM turnover frequencies (TOF) up to 10,000 mol/mol Rh/h with the cooperative catalyst when the reaction is conducted continuously in a gas–liquid segmented flow reactor at moderate temperature and pressure (115 °C and 28 bar). The segmented flow refers to a regular train of alternating gas–liquid segments continuously moving along the flow reactor, which is commonly referred to plug flow format (i.e., gas plugs surrounded by a continuous liquid phase)^22^. The synergistic effect of the developed cooperative catalyst system with the heat and mass transfer advantages of the segmented flow reactor allows for the HAM reaction with sterically hindered amines at good to excellent yields (71–92%) in less than 3 h with 0.1 mol% Rh(acac)(CO)_2_. Our mechanistic investigations reveal that the HAM rate acceleration results from the cooperation between the Rh/N-Xantphos and the benzoic acid to lower the energy barrier for the H–H bond cleavage to form the Rh hydride needed to drive the enamine hydrogenation. Rapid enamine hydrogenation drives the equilibrium-limited condensation with α-branched amines in the forward direction and avails Rh to catalyze the olefin hydroformylation. Gas–liquid segmented flow format enhances the hydrogen transfer rate into the liquid phase and maximizes the concentration of the active Rh–H species. We demonstrate that the gas–liquid segmented flow reactor can enable selective acceleration of the slow elementary step in the HAM reaction, which is the enamine hydrogenation, but not the undesired aldol condensation. Through reaction condition optimization, we extended the substrate scope of HAM to alkyl and aryl amines as well as primary amines and styrenes. Another unique aspect of the developed cooperative catalyst is its recyclability. Leveraging the ionic nature of the cooperative catalyst system, we demonstrate catalyst precipitation and recycle for up to 4 cycles. Additionally, through on-the-fly solvent switching from methanol/toluene mixture to toluene we present an on-demand selectivity switching from HAM to hydroaminovinylation without the need for ligand replacement. The developed cooperative catalyst in combination with the scalable segmented flow reactor can facilitate decentralized continuous manufacturing of a wide range of APIs and fine chemicals through the atom-efficient HAM reactions with a high degree of flexibility and catalyst recyclability.

## Results

### Reaction optimization in flow

**Fig. 2:**
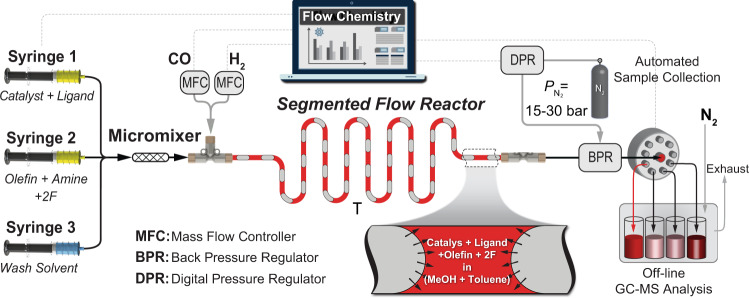
Schematic illustration of the flow chemistry platform utilized for the accelerated HAM reactions. The sample collection chamber allows for liquid sampling at different reaction (residence) times by varying the total feed flow rate. MFC: Mass Flow Controller, BPR: Back Pressure Regulator, DPR: Digital Pressure Regulator, 2F: 2-fluoro-4methylbenzoic acid.

We began our investigations of the HAM synthetic route by studying the effect of ligand (L) and co-catalyst on the HAM of 1-octene with piperidine to form N-nonylpiperidine (**1a**) using a more economic catalyst precursor Rh(acac)(CO)_2_ instead of Rh(cod)BF_4_. We developed a gas–liquid segmented flow reactor, shown in Fig. 2, for in-flow studies of the HAM reactions (see “Methods”, General Procedure 1).

Seeking to address the aforementioned challenges of BISBI and Xantphos ligands in HAM reactions and inspired by the cooperative hydrogenation work of de Bruin and co-workers^23^, we wondered if a benzoic acid co-catalyst in combination with the enhanced mass transport rate of the gas–liquid segmented flow reactor could provide a synergistic effect to accelerate the kinetics of HAM reactions. The co-catalyst screening was carried out with bidentate phosphine ligands because of the ligand previous success in HAM of olefins^18,19^. Table 1 presents a summary of the ligand and co-catalyst screening experiments of HAM of 1-octene. Xantphos resulted in an amine yield of 6.6% after 15 min residence (reaction) time (Table 1, entry 1). Higher yield and *l*/*b* were obtained with BISBI ligand. However, the yield of the unreacted aldehyde and enamine remained significant (Table 1, entry 2). Changing the substitution on the phosphorous from phenyl to alkyl or amine did not result in an enhanced activity (Table 1, entries 3 and 4). Switching to N-Xantphos ligand increased the amine yield by 4 times to reach 28.8%. However, a significant portion of the substrate were accumulated as the enamine intermediate, indicating that the overall HAM transformation is limited by the enamine hydrogenation step (Fig. 1a, step iii). N-Xantphos, in comparison with Xantphos, increased the amine *l*/*b* ratio from 50 to 98 and the enamine to aldehyde ratio from *ca*. 2 to 5 (Table 1, entry 4), indicating the superior performance of N-Xantphos in terms of hydroformylation linear selectivity and enamine hydrogenation activity. Without any co-catalyst addition, the only side product observed were cis- and trans- 2 octene.

**Table 1:** Ligand and co-catalyst screening of HAM reaction in segmented flow.


Entry	L	Co-catalyst	Amine yield (%)	Amine (l/b)	1-octene conv. (%)	Ald. yield (%)	Enamine yield (%)
**1**		–	6.6	50	83	17	34.9
**2**		–	12.2	>100	91.6	8.8	54.5
**3**		–	6.3	27	8.8	0	2.5
**4**		–	0	–	0.1	0	0.1
**5**		–	28.8	98	91.7	9.0	43
**6**			71.8	54.8	90.2	0	4.5
**7**			88.3	84	94.1	0	2.2
**8**^a^			46.7	73	55.8	0	3.8

^a^Reaction is conducted in a batch reactor.

Interestingly, addition of 0.5 mol% of the co-catalyst, 4-trifluoromethylbenzoic acid, to the Rh/N-Xantphos catalyst increased the amine yield to 71.8% and decreased the yield of the aldehyde and enamine intermediates down to zero and 4.5%, respectively (Table 1, entry 6). This result indicates a catalytic effect of the benzoic acid additive on either the condensation or the hydrogenation step (Fig. 1a, steps ii and iii). However, introduction of the co-catalyst decreased the amine *l*/*b* selectivity from 97 to 55. Building on the superior performance of the Rh/N-Xantphos system in the presence of the co-catalyst, in the next set of experiments we explored the effect of the co-catalyst structure on the HAM reaction (Supplementary Table 1). High-throughput flow screening of a rationally selected library of 10 different benzoic acids unveiled the fluorine substitution at the ortho position of the benzoic acids results in the highest amine *l*/*b*. This result could be attributed to the formation of the less sterically hindered Rh carboxylate species that contribute to the pro-branched hydroformylation without fluorine substitution at the *ortho* position. In particular, the highest amine yield was achieved with 2-fluoro-4-methylbenzoic acid **2F** (Table 1, entry 7).

To demonstrate the rate acceleration by the segmented flow reactor, in a separate experiment, we conducted the HAM reaction in a batch reactor using the co-catalyst condition of entry 7 in Table 1. The batch HAM reaction (Table 1, entry 8) resulted in a significantly lower 1-octene conversion, amine yield, and amine *l*/*b* selectivity compared to the segmented flow reactor. The higher 1-octene conversion and amine/enamine ratio in the flow reactor supports the accelerating effect of the gas–liquid segmented flow format on the tandem hydroformylation and hydrogenation steps (Fig. 1a, steps i and iii).

**Fig. 3:**
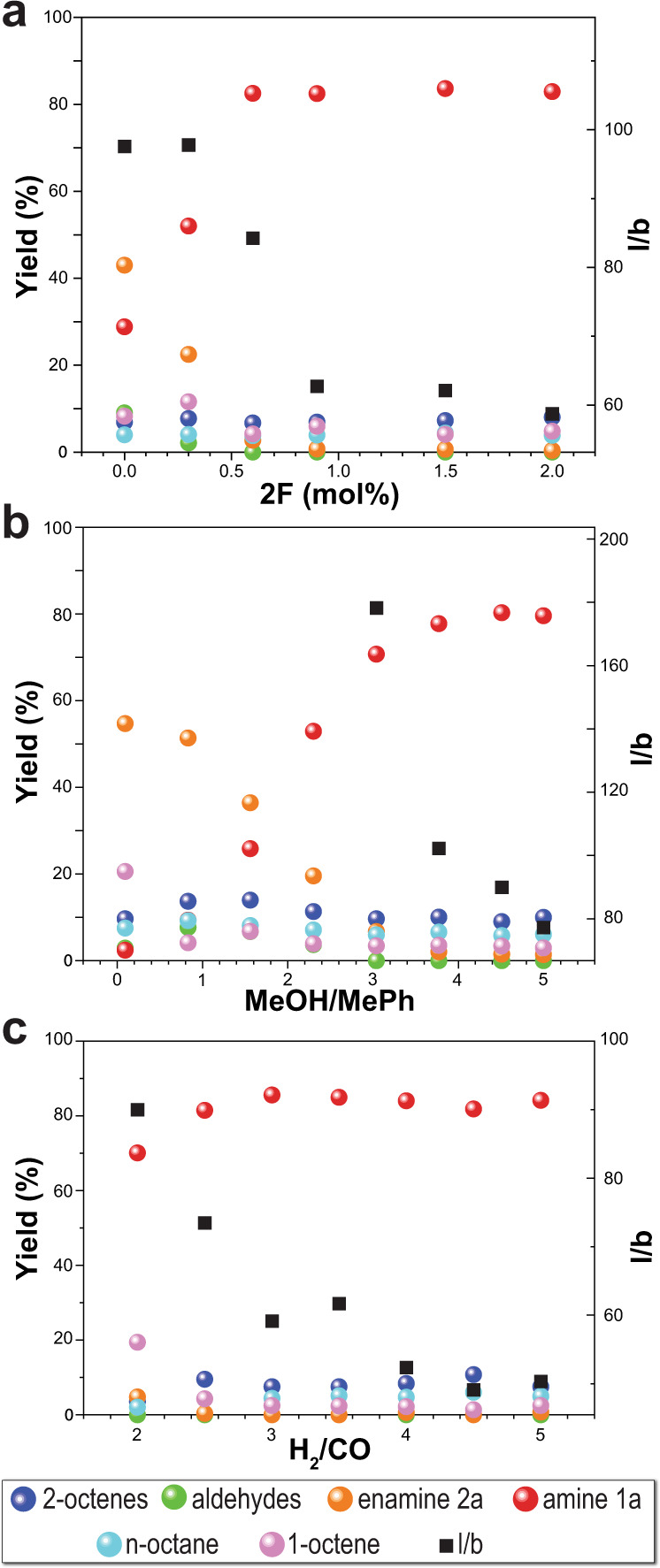
In-flow reaction optimization. Optimization of the HAM reactions for **a** 2F loading at MeOH:MePh volume ratio of 5 and H_2_:CO ratio of 3.5, **b** MeOH:MePh volumetric ratio at 2 mol% 2F and H_2_:CO ratio of 3.5, and **c** H_2_:CO ratio at 2 mol% 2F and MeOH:MePh volume ratio of 4. General HAM reaction conditions: 125 °C, 15 min residence time, 24 barg total pressure, 3.5 gas:liquid volumetric ratio, 0.45 M 1-octene, 0.1 mol% Rh(acac)(CO)_2_, and 0.4 mol% N-Xantphos.

After identifying N-Xantphos and **2F** as the optimal ligand and co-catalyst for the proposed accelerated HAM synthetic route, we studied the effect of the co-catalyst loading as well as the solvent and gas composition on the amine yield and selectivity. This systematic study (Fig. 3) allowed us to investigate and elucidate the effect of each reaction parameter on the three elementary steps of the HAM reaction; hydroformylation, condensation, and hydrogenation. The amine yield monotonically increased with increasing **2** **F** loading from zero to 0.6 mol% to reach a maximum of 86%, while the amine *l*/*b* ratio decreased from 117 to 59. The increase in the amine yield was accompanied by a decrease in the aldehyde yield and enamine intermediates (0% at 0.6 mol% and 0.9 mol% of **2F**), shown in Fig. 3a. No evidence of benzoic acid esterification or hydrogenation was observed at the highest **2F** loading. The solvent composition also has a significant impact on the HAM reaction when **2F** is added at 2 mol%. In the absence of methanol (i.e., toluene only), both hydroformylation and enamine hydrogenation (Fig. 1a, steps i and iii) were slow as indicated by the incomplete conversion of 1-octene (79%), and the high yield of the enamine (55%), shown in Fig. 3b. Gradual increase in the methanol volumetric ratio results in suppression of the unreacted 1-octene and enamine (0% unreacted 1-octene and enamine at the methanol:toluene volumetric ratio of 3.77). The aldehyde yield increases with increasing methanol content and reaches a maximum at methanol:toluene volumetric ratio of 0.83, before it decreases to 0% at methanol:toluene volumetric ratio of 3. The increase in the aldehyde yield can be attributed to the acceleration in the rate of hydroformylation at low methanol content and is reversed by the acceleration in the rate of condensation at the high methanol content. Interestingly, no branched product was observed at the low methanol content up to methanol:toluene volumetric ratio of 2.3. Although further increasing of the volumetric ratio of methanol increases the amine yield, it results in a lower amine *l*/*b* ratio. Increasing the methanol:toluene volumetric ratio beyond 3.7 does not result in an increase in the amine yield but reduces the amine *l*/*b*. Furthermore, varying the reaction solvent composition did not affect the yield of n-octane and 2-octene isomers.

The results shown in Table 1 suggest that the HAM reaction is limited by the enamine hydrogenation. Since the required stoichiometric ratio of H_2_ to CO for a complete HAM transformation is 2, in the next set of experiments we studied the effect of H_2_:CO ratio from 2 to 5, while maintaining the total pressure at 24 barg. At H_2_:CO ratio of 2, an incomplete conversion of 1-octene was achieved (Fig. 3c). Increasing the partial pressure of H_2_ (H_2_:CO = 3) resulted in an increase in both 1-octene conversion and amine yield from 70 to 85% at 2 mol% loading of **2F**. Further increase of the H_2_:CO ratio resulted in an increase of the 2-octenes yield, while decreased the amine *l*/*b* to 50. The results of the gas composition screening, shown in Fig. 3c, shows the H_2_:CO ratio of 3–4 achieves the best combination of high amine yield and reasonably high amine l/b (>20).

**Fig. 4:**
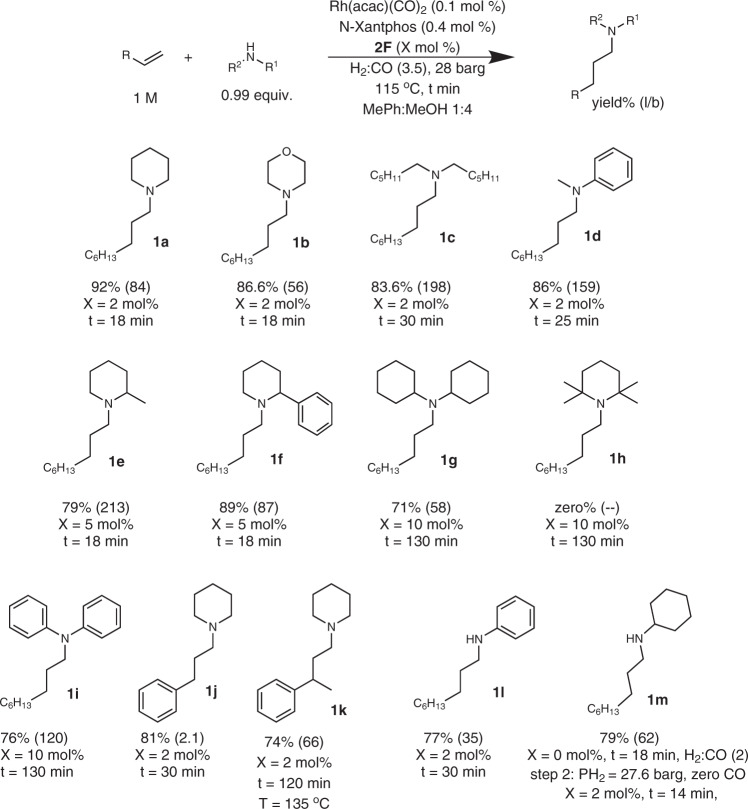
In-flow HAM substrate scope screening with the cooperative catalyst. Rh and N-Xantphos loading were 0.1 and 0.4 mol%, respectively. For each in-flow HAM reaction, 4 ml of the effluent liquid was collected, and the product was isolated and purified for analysis. The reported yields are the isolated yields, and the l/b values are measured by gas chromatography–mass spectrometry (GC-MS).

In order to maximize the flow reactor throughput when continuously conducting the HAM reaction, we increased the concentration of the olefin substrate to 1 M, the pressure to 28 barg, and the gas:liquid volumetric ratio to 5; The gas:liquid volumetric ratio was increased to 5 to provide excess CO gas relative to the olefin and suppress *n*-octane formation. In order to suppress the amine yield loss due to olefin isomerization to 2-octene and hydrogenation to *n*-octane, we decreased the reaction temperature from 125 to 115 °C and achieved an amine yield of 92% (Figs. 4, 1a).

### Substrate scope

With the optimized conditions in hand, we next investigated the scope of the accelerated HAM reaction with the cooperative catalyst in flow (Fig. 4). Specifically, in the substrate scope study, we focused on more challenging a-branched amines. Using the optimized cooperative catalyst, continuous synthesis of N-nonylpiperidine (1a) at 0.93 mmol/h was achieved with in only 18 min residence time (92% yield). Morpholine was also alkylated in 18 min residence time to produce **1b** with a yield of 86.6%. The residence time needed to alkylate the acyclic dihexylamine with the cooperative catalyst to obtain **1c** at 83.6% yield had to be increased to 30 min to account for the slower hydroformylation observed with this amine. For comparison, the batch alkylation of dihexylamine under similar reaction conditions to the in-flow HAM with the cooperative catalyst resulted in only 48% yield and amine *l*/*b* of 13 after 4 h. This result further supports the superior performance of the segmented flow reactor in promoting both HAM reactivity and selectivity. HAM of anilines results in products and intermediates commonly applied in pharmaceuticals, plastics, and rubber additives, as well as OLEDs^1–5^. In-flow HAM of N-methylaniline with the cooperative catalyst resulted in 86% yield of **1d** and *l*/*b* of 159. The more sterically hindered 2-methylpiperidine was successfully alkylated in the segmented flow to give 79% yield of **1e** and *l*/*b* of 213. The **2F** loading was increased from 2 to 5 mol% to accelerate the relatively slow reaction with the hindered enamine. Under these conditions, no aldol products were detected, which further supports the effectiveness of **2F** in selectively catalyzing the reductive amination with the hindered amine over the aldehyde self-condensation. For comparison, batch alkylation of 2-methylpiperidine under similar conditions to the flow reactor resulted in 74% yield of **1e** with *l*/*b* of 11 after 24 h reaction time. This result of batch vs. flow HAM of the hindered amines illustrates the effectiveness of the segmented flow reactor in promoting both the HAM reactivity and selectivity for the sterically hindered amines.

The substrate scope was then successfully extended to 2-phenylpiperidine with a 89% yield of **1f** and *l*/*b* of 87 in only 18 min, using 5 mol% of the co-catalyst **2F**. Encouraged by the results obtained with the slightly hindered 2-substituted piperidines, we moved to the more hindered dicyclohexylamine. To our delight, dicyclohexylamine was successfully alkylated at 71% yield of **1** **g** with 10 mol% of the co-catalyst **2F** at the residence time of 130 min in the segmented flow reactor. To the best of our knowledge, this is the only HAM report of hindered amines at this high rate and selectivity. Attempting to extend the scope to 2,2,4,4-tetremethylpiperidine failed (no **1** **h** product was formed) and resulted in the accumulation of the unreacted aldehyde.

Phenylaniline was also alkylated in flow with the cooperative catalyst (72% yield of **1i** with *l*/*b* of 160). For this relatively hindered, less basic substrate, **2F** loading had to be increased to 10 mol % and the residence time to 130 min. Next, HAM of piperidine with styrene was performed at 81% yield (**1j**) and *l*/*b* of 2.1. The relatively low *l*/*b* selectivity for product **1j** is attributed to the stabilization of the benzylic Rh species induced by the η2 electron donation from the arene ring^24^. Continuous HAM of the disubstituted olefin, α-methylstyrene, was accomplished with the cooperative catalyst at an increased temperature (135 °C) and residence time (120 min), resulting in a 74% yield of **1k** and *l*/*b* of 66. Additional in-flow HAM optimization of the primary amine, aniline, revealed that a higher yield can be obtained when **2F** loading is lowered to 1 mol % to suppress the double alkylation of the amine. At this lowered loading of the co-catalyst, the residence time in the segmented flow reactor was increased to 30 min to obtain a 77% yield of the monoalkylated product **1l** and *l*/*b* of 35. Next, we investigated in-flow HAM with cyclohexylamine. Our in-flow screening experiments revealed that the optimal HAM of 1-octene with cyclohexylamine can be achieved when the reaction is carried out in two tandem steps with the in-line addition of the co-catalyst **2F** for the second step. The H_2_:CO ratio was maintained at 2 for the first step to selectively form the imine N-nonylcyclohexylimine, while the second step was performed under 26 barg of H_2_ for 14 min to obtain the desired monoalkylated product **1m** with a 79% yield and *l*/*b* of 62. In-flow HAM of carbazole could not be achieved because of the ultra-low solubility of carbazole in the reaction solvent used in this study at room temperature. Moreover, ammonia could not be alkylated under the developed HAM synthetic route because of the slow imine hydrogenation relative to condensation side reactions. The intramolecular HAM of *o*-isopropenylaniline resulted in low yield of the desired product, 4-methyl-1,2,3,4-tetrahydroquinoline, because of the competing intermolecular reaction.

### Catalyst recycling

**Fig. 5:**
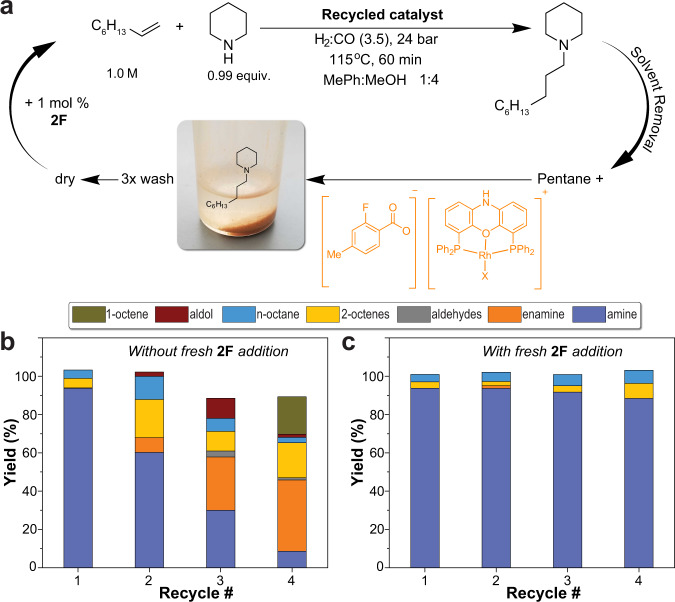
Catalyst recycling scheme. **a** Schematic of the developed catalyst recycling process. HAM performance of the Rh/N-Xantphos catalyst recycled without (**b**) and with **c** the addition of 2F.

Owing to the high cost of Rh catalysts, it is necessary to maximize the productivity of the catalyst before sending for precious metal reclaiming^25,26^. During the product isolations of the in-flow HAM reactions, we observed that an orange solid precipitates upon solvent removal from the crude product mixture under vacuum. In most cases, the precipitated solids were insoluble in the product amine. Upon addition of pentane to the residue after solvent removal, the powder precipitates and can be separated by settling or centrifugation. Additional washing of the powder with more pentane followed by pentane removal allows for complete separation of the product amine from the powder, as shown in Fig. 5a. To assess the HAM reactivity of the isolated powder, the solid precipitate was dried under vacuum and then dissolved in the reaction solvent (MeOH:MePh volumetric ratio of 4:1) at room temperature under inert atmosphere. Fresh reactants were then added to the powder, and the HAM reaction was performed under the same reaction conditions. A rapid decay in activity was observed over the first three recycles (Fig. 5b). Both yields and *l*/*b* selectivity of amine **1a** decreased after catalyst recycling (from 93% yield to less than 10% and *l*/*b* of 60 to 5), while the yield of the enamine **2a** and the aldol side products increased. Incomplete consumption of 1-octene was also observed after the third catalyst recycling. The loss in activity was first observed in the hydrogenation step, followed by the hydroformylation step. We postulated that the observed decrease in the HAM activity after the catalyst recycling was due to the loss of the co-catalyst **2F** during the recycling process. To validate this hypothesis, we replenished the reaction mixture with fresh 1 mol% of **2F** following each recycle round. When catalyst recycle was performed with the addition of 1 mol % of **2F**, an excellent recyclability and activity of the catalyst system was achieved, Fig. 5c. The amine yield and *l*/*b* using the developed HAM synthetic route and catalyst recycling process were maintained higher than 85% and 55, respectively, after three rounds of catalyst recycles. We attribute the enhanced recyclability of the Rh/N-Xantphos catalyst upon the addition of the co-catalyst **2F** to stabilization of the ionic Rh carboxylate complex (Fig. 5), that is insoluble in long-chain, low polarity amines. Furthermore, the fluorine substitution on **2F** could also contribute to the catalyst recyclability by minimizing the solubility of the Rh complex in the product amines. The Rh loss to the amine product was found to be less than 5% of the initial mass of Rh (measured by ICP-MS). This concept has previously been utilized to synthesize fluorinated ligands that can facilitate Rh extraction from organic media in hydroformylation reactions^27^. The presence of the co-catalyst **2F**, in addition to reducing the catalyst solubility, enhances the stability of the ionic species when exposed to air, as evident by the persistence of the orange color of the powder when fresh **2F** was added after each round of the catalyst recycle, that is contrary to the change in the powder color to pale green without fresh **2F** addition. Recycling of Rh from amination solutions are less explored in the literature^20,21,28^. This study illustrates the effectiveness of a commercially available co-catalyst in accelerating the HAM reaction and allowing for catalyst recycling at the same time.

### On-demand selectivity switching to enamine

**Fig. 6:**
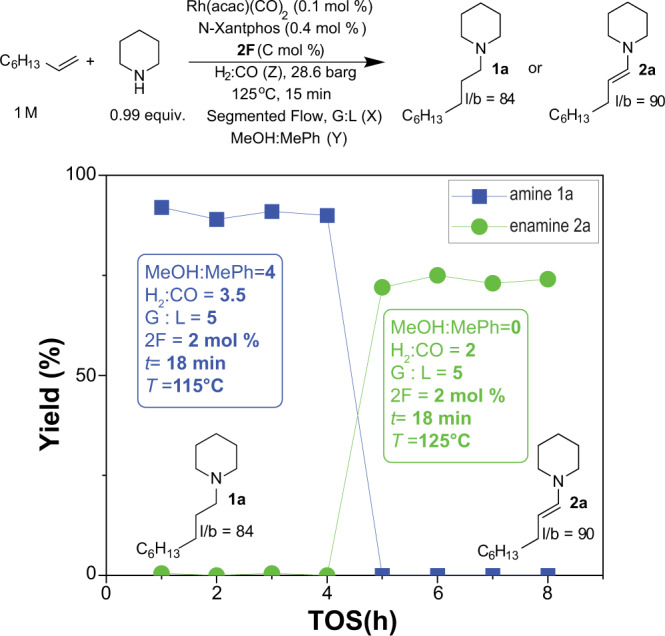
Online reaction product switching. On-the-fly selective switching from the amine to enamine formation with Rh/N-Xantphos in the presence of the co-catalyst 2F in the segmented flow reactor. G:L is gas to liquid volumetric feed ratio, *t*: residence time.

Enamines are nucleophilic reactants that can undergo alkylation or acylation with alkyl halides and acyl halides, respectively, and thus are widely used in organic synthesis^29^. Selective formation of enamines by condensation of an aldehyde with an amine often requires the slow addition of the aldehyde or the addition of the amine in large excess to suppress the aldehyde self-condensation. The hydroaminovinylation of the olefin with the amine is an alternative strategy that allows for the one-pot, auto-tandem hydroformylation-condensation route that suppresses the formation of the undesired aldol. The main limitation in this strategy is that the formed enamine often undergoes hydrogenation under the hydroformylation conditions. The ligand NAPHOS was shown to be more selective to enamine formation than Xantphos in toluene at 65 °C with enamine *l*/*b* selectivity exceeding 90^30^. However, the reaction required 16 h to reach completion. More recently, hydroaminovinylation was shown to be driven to completion in 1 h at 130 °C in solvent-free conditions with a diphosphite ligand, but with enamine *l*/*b* values lower than 25^31^. To address these limitations, we explored the performance of N-Xantphos ligand in the hydroaminovinylation of 1-octene with piperidine in flow under the optimized HAM conditions developed in this work. Without the co-catalyst **2F**, the yield of enamine **2a** increased from 0 to 32%. However, the yield of amine **1a** remained high at 42% (Supplementary Fig. 1). Lowering the reaction temperature to 95 °C and the H_2_:CO ratio to 1 did not completely suppress **2a** hydrogenation to **1a**, but drastically reduced the hydroformylation reactivity (less than 15% yield of **2a** + **1a**). Through our solvent screening experiments, we discovered that on-the-fly solvent switching to 100% toluene could suppress **2a** hydrogenation and maintain **2a** yield at 55% with the remaining yield being unreacted aldehyde and olefins. Increasing the co-catalyst loading to 2 mol% and reaction temperature to 125 °C, further increased the yield of **2a** to 74% with an enamine *l*/*b* of 90 (Supplementary Fig. 1). Inspired by this finding, we demonstrated the unique ability of the flow reactor to switch on-demand from HAM synthetic route to hydroaminovinylation (Fig. 6) with the same cooperative catalyst (N-Xantphos ligand and **2F**).

### Mechanistic study

Next, we investigated the mechanism by which the cooperative catalyst (Rh/N-Xantphos +**2F**) rapidly catalyzes the HAM. The Rh-catalyzed hydrogenation of enamine **2a** was performed with N-Xantphos and Xantphos ligands to compare the hydrogenation activity of the two ligands under CO/H_2_ atmosphere (Table 2). The reaction was performed at 105 °C and 20 barg to slow down the hydrogenation and allow for accurate reactivity comparison in batch and flow reactors. The yield of the amine product with N-Xantphos was 39% which is 3 times more than the yield obtained with Xantphos after 30 min in the batch reactor (Table 2, entries 1 and 2). The higher hydrogenation activity with N-Xantphos could be attributed to the electron donating character of the –NH moiety^32^, that can lower the barrier to the oxidative addition of the H–H bond. Addition of 2 mol% of the co-catalyst **2F** resulted in an enhanced hydrogenation activity with both ligands (Table 2, entries 3, and 4). This result suggested that the accelerating effect realized by **2F** is not due to protonation of the –NH moiety on the N-Xantphos by the acid. Indeed, the ^1^H and ^31^P NMR shifts of the ligand N-Xantphos in deuterated methanol/toluene solvent are essentially unchanged when the acid **2F** or the amine dicyclohexylamine are added (see Supplementary Table 2). It is worth noting here that deprotonation of the ligand N-Xantphos by a strong base resulted in an improved activity towards oxidative addition to aryl chlorides^33^. However, it is unlikely that this effect occurs under the less basic HAM conditions as supported by the NMR data in Supplementary Table 2. The amine yield by addition of the co-catalyst **2F** was increased to 99% in only 10 min of the reaction time, when the reaction was performed in the segmented flow reactor (Table 2, entry 5).

**Table 2:** Enamine hydrogenation activity of N-Xantphos vs. Xantphos with and without 2F.


Entry	L	Y (mol %)	Reactor	1a yield (%)	Unreacted 2a (%)
**1**	N-Xantphos	0	30 min Batch	39.4	58
**2**	Xantphos	0	30 min Batch	12.2	83.1
**3**	N-Xantphos	2	30 min Batch	46.4	51.7
**4**	Xantphos	2	30 min Batch	26.9	68.7
**5**	N-Xantphos	2	10 min Flow	98.8	1.2

Contrary to hydrogenation, there is no catalytic effect of **2F** on piperidine condensation with n-nonanal in the absence of Rh catalyst; the yield of enamine **2a** slightly decreased from 64 to 58% upon **2F** addition to a 60 min batch condensation reaction (Supplementary Table 3). The decrease in the enamine yield was accompanied by an increase in the C_18_ aldol products (from *ca*. 12 to 22%). The boost in the desired reaction was only realized when **2F** was added in the presence of Rh catalyst which resulted in an increase in the combined yield of **1a** and **2a** to 92% and decreased the aldol yield to less than 5% (Supplementary Table 3). The same observation applies to the condensation/reductive amination of the sterically-hindered dicyclohexylamine with n-nonanal. Without Rh catalyst, the combined yield of the amine and enamine remained at 3% with and without the co-catalyst **2F** (Supplementary Table 4, entries 1 and 2). Addition of the Rh/N-Xantphos catalyst increased the amine yield to 20% (Supplementary Table 4, entry 3). The increase in the amine yield is because the condensation step is equilibrium-limited, and the Rh addition helps drive the condensation equilibrium in the forward direction by hydrogenating the enamine product. The combined yield almost doubled to 41% when both **2F** and Rh/N-Xantphos catalyst were added (Supplementary Table 4, entry 4). This result further supports the cooperative effect of the co-catalyst and Rh/N-Xantphos on catalyzing the enamine hydrogenation and thus driving the condensation equilibrium forward.

**Fig. 7:**
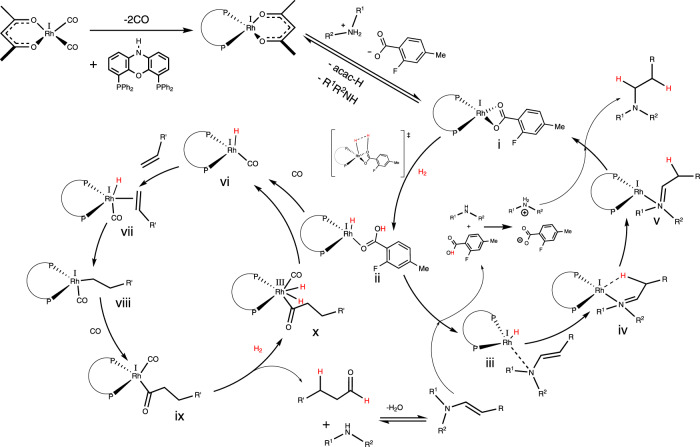
Proposed reaction mechanism. Proposed mechanism of the HAM reaction with the cooperative catalyst developed in this work.

Following the identification of the cooperative effect of **2F** with Rh/N-Xantphos on catalyzing the reductive amination, we proceeded with hydrogen-deuterium (H/D) scrambling experiments. The H/D ratio of the hydrogenation step represents the kinetic isotope effect on the reductive amination. Changing the gas from H_2_ to D_2_ decreased the % hydrogenation from 50.2 to 39.1% (Table 3, entries 1 and 2). The H/D ratio was found to be 1.28, indicating that the hydrogenation reaction could be limited by a hydride formation or hydride delivery step, but not the protonation of the Rh-aminyl complex or the enamine coordination (see Fig. 7 for the proposed mechanism). The decrease in the H/D ratio to 1.18 in the presence of the co-catalyst indicates the lower hydridic nature of the rate-limiting step upon the addition of **2F**. The same trend was observed in the reductive amination with the less hindered piperidine, where the measured H/D ratio of the dehydrogenation decreased from 1.13 to 1.03 upon the addition of the co-catalyst **2F** (Supplementary Table 5).

**Table 3:** H/D effect on the reductive amination of dicyclohexylamine with 2F + Rh/N-Xantphos.


Entry	Y (mol %)	Gas	2g Yield (%)	1g yield (%)	Hydrogenation (%)^a^	H/D (% hydrogenation)
1	0	H_2_	1.34	1.35	50.2	1.28
2	0	D_2_	1.4	0.9	39.1	1.28
3	2	H_2_	1.15	2.75	70.5	1.18
4	2	D_2_	0.97	1.45	59.9	1.18

^a^Calculated as (1g/(1g + 2g)%.

The olefin hydroformylation reactivity was not affected by **2F** addition or switching between H_2_ and D_2_ (Supplementary Table 6). This result suggests that the hydroformylation with Rh/N-Xantphos ligand is not limited by hydride formation or delivery as indicated by the H/D ratio of 1.03. Earlier studies with N-Xantphos showed that the hydroformylation is limited by olefin coordination or CO dissociation^32^, which is in agreement with the results shown in Supplementary Table 6. The hydroformylation experiment was performed without amine addition to eliminate the effect of enamine hydrogenation on the hydroformylation reactivity. It should be noted that the N-Xantphos ligand has been shown to be slower than Xantphos in the hydroformylation of 1-octene under typical hydroformylation conditions^32^. However, the N-Xantphos is faster in 1-octene hydroformylation under the HAM synthetic route developed in this study. This dichotomy is attributed to the faster enamine hydrogenation by N-Xantphos, and thus promoting the Rh catalyst to catalyze olefin hydroformylation under this HAM synthetic route. Same explanation applies to the indirect accelerating effect of **2F** on the hydroformylation rate under HAM conditions where the co-catalyst accelerates the enamine hydrogenation, resulting in enhanced availability of Rh for the hydroformylation step of the HAM synthetic route. From our results of the H/D scrambling and control experiments, we propose that the HAM reaction, developed in this work, proceeds via the formation of Rh/N-Xantphos-**2F** complex **i** formed form the loss of CO and the deprotonation of **2F** by the amine reactant, as shown in Fig. 7. The heterolytic cleavage of the H-H bond is believed to be more facile than the homolytic oxidative addition that results in Rh dihydride. Similar effect has been proposed for Co^23^, Ni^34^, and Ru^35^ catalysts. Ligand exchange between **2F** and the enamine results in the formation of the Rh hydride amine complex **iii** that can undergo hydride transfer to the unsaturated C=C bond. *P*-methyl carboxylic acids are less acidic than their unsubstituted analogs, and thus should undergo a more facile transition from species **i** to **ii** in the proposed reaction mechanism. This is also supported by the higher amine/enamine ratio by **2F** in Supplementary Table 1.

Protonation of the formed Rh amine complex **v** by the ammonium cation results in the formation of the product amine and the Rh-**2F** complex **i**. From the H/D scrambling experiments, it is reasonable to propose that the rate-limiting step is either the cleavage of the H–H (formation of **ii**) bond or the hydride delivery (formation of **iv**) in the enamine hydrogenation. The need for high H_2_ pressure to drive the enamine hydrogenation in combination with the comparable hydrogenation obtained for the less sterically hindered amine **1a** and the more sterically hindered amine **1g** suggest that the oxidative addition of the H–H bond is the hydrogenation rate-limiting step^36^. The hydroformylation cycle proceeds via the formation of the rhodium carbonyl hydride vi followed by the olefin coordination, and the hydride addition to form complexes vii and viii, respectively. CO migratory insertion results in the formation of the acyl complex **ix** that can perform the oxidative addition of H–H bond followed by the reductive elimination to produce the aldehyde and the Rh hydride vi. The aldehyde condensation with the amine is not often regarded as a metal-catalyzed reaction, and thus the ligand structure does not play a role in this step. Instead, solvent polarity and medium acidity are often increased to catalyze this step^37,38^. However, the condensation of the aldehyde with sterically hindered amines is equilibrium-limited^39^, and thus, fast enamine hydrogenation results in faster condensation relative to the aldehyde aldol condensation, as demonstrated by the cooperative catalyst system developed in this study.

Under HAM conditions, both hydroformylation and enamine hydrogenation occur simultaneously and share the available Rh catalyst, and thus their rates are expected to be lower than the rates obtained when each of the two reactions is performed separately. To measure the TOFs of both reactions that can be achieved in flow under typical HAM conditions, the catalyst loading was reduced from 0.1 to 0.01 mol% and the 1-octene reaction with piperidine was performed under variable residence times (ligand to Rh ratio at 4 and co-catalyst **2F** loading at 2 mol %, at 115 °C and gas to liquid of 5). The residence (reaction) time was varied by tuning the total volumetric flow rate. The initial hydroformylation TOF was 9000 mol/mol Rh/h, while the HAM TOF was 4000 mol/mol Rh/h (Supplementary Fig. 2a). To the best of our knowledge, this is the highest HAM TOF reported up to date. The HAM TOF could be further boosted in flow by increasing the gas to liquid volumetric ratio. High gas to liquid volumetric ratio results in smaller liquid segments that exhibit shorter H_2_ diffusion length. The enhanced diffusion results in maximization of the Rh-hydride active species ii that catalyze enamine hydrogenation which promotes the Rh catalyst for catalyzing the hydroformylation step. When the gas to liquid volumetric ratio is 10, the HAM TOF approaches 10,000 mol amine/mol Rh/h (Supplementary Fig. 2B). While an ultra-high TOF can be achieved at high gas to liquid volumetric ratios, the product throughput decreases. The choice of the optimum gas to liquid volumetric ratio and Rh loading in HAM segmented flow reactors depends on process economics.

## Discussion

In summary, we introduced the fastest Rh-catalyzed hydroaminomethylation process known to date by leveraging a synergistic effect of the cooperative Rh/N-Xantphos and the co-catalyst (fluorinated benzoic acid **2F**), and the enhanced gas–liquid mass transfer in the segmented flow reactor as an enabling factor. The process furnishes cyclic and acyclic alkylamines, anilines, and morpholines in high yield and regioselectivity, 70 times faster than the state-of-the-art Xantphos-ligated Rh catalyst in a batch reaction. The HAM scope was expanded to include primary amines without the need for Ir hydrogenation catalyst and included hindered amines, such as alkylated dicyclohexylamine. Rapid enamine hydrogenation is the key feature in the developed HAM synthetic route and is enabled by the cooperative effect of the co-catalyst and Rh/N-Xantphos that facilitates the oxidative addition of the H–H bond to Rh. The rapid enamine hydrogenation drives the condensation equilibrium forward and enables the HAM of hindered amines over the undesired aldol products. Leveraging the ionic nature of the catalyst system (Rh/N-Xantphos and **2F**), we developed a robust recycle strategy of the increasingly expensive Rh metal and achieved TOF values exceeding 10,000 mol amine/mol Rh/h. On-the-fly tuning of the flow reactor conditions, enabled on-demand switching of the product selectivity from the amine to the enamine. The unique online reaction product switching from amine to enamine combined with the recyclability of the developed cooperative catalyst illustrates unique capabilities of this catalyst system for continuous manufacturing of hindered amines through the atom-efficient HAM route. The accelerated in-flow production of hindered amines through the scalable hydroaminomethylation synthetic route, developed in this work, will facilitate on-demand/on-site synthesis of a wide range of specialty compounds in chemical and pharmaceutical industries. Furthermore, the proposed catalyst cooperativity and recycling protocol of the N-Xantphos ligated precious metals with **2F** is amenable to automation and will find applications in other challenging homogeneous catalytic reactions.

## Methods

### General Procedure 1: HAM in the segmented flow reactor

The catalyst, dicarbonyl 2,4-pentanedionato rhodium(I), and the ligand, 4,6-Bis(diphenylphosphino)-10*H*-phenoxazine (N-Xantphos), were dissolved in toluene under inert atmosphere inside a glovebox. Methanol was then added to dilute the catalyst/ligand solution to the target solvent composition. An 8 mL-stainless steel syringe connected to a Teflon tubing (fluorinated ethylene propylene (FEP), inner diameter (ID): 0.01″, outer diameter (OD): 1/16″) was filled with the catalyst and ligand solution under inert atmosphere. Another 8 mL-stainless steel syringe connected to a 0.01″ FEP tubing was filled with a mixture of the olefin, the amine, and the co-catalyst dissolved in methanol/toluene solvent under inert atmosphere. The syringe outlets were capped under inert atmosphere with Teflon screw caps (IDEX Health & Science) before being transferred outside of the glovebox and connected to a PEEK cross connection (IDEX Health & Science), where the solutions from the syringes were mixed at a pre-selected volumetric ratio before contacting the gas. During the ligand and co-catalyst screening experiments (results shown in Table 1 and Supplementary Table 1), an additional 8 ml stainless steel syringe was utilized to separately load the ligand or co-catalyst, thereby providing additional flexibility for online tuning of the co-catalyst and ligand loading. Gas–liquid segmentation was achieved by contacting the combined liquid stream with the gas flow in a stainless steel T-junction (1/8″ OD, Swagelok) before entering the stainless-steel flow reactor (1/8″ OD, 1/16″ ID, and 94 cm length). Liquid flowrates were controlled by syringe pumps (Harvard PHD ULTRA) and gas flowrates were controlled by individual mass flow controllers (EL-Flow^®^, Bronkhorst). The residence time is calculated on cold basis using the flow reactor volume and the feed flow rates. Time-to-distance transformation was used to vary the residence (reaction) time by tuning the total volumetric flow rate at the same gas:liquid feed ratio. The flow reactor temperature was controlled through a hotplate and oil bath with a temperature probe immersed in the oil bath. The flow reactor pressure was controlled with a nitrogen (N_2_) pressure connected to the control port of a backpressure regulator (Equilibar) integrated at the outlet of the flow reactor coil. The flow reactor effluent was passed through a 10-way selector valve (VICI, EUHB) and directed to a custom-designed waste collection chamber equipped with an exhaust line for unreacted carbon monoxide (CO) and hydrogen (H_2_) removal. Prior to each in-flow hydroaminomethylation (HAM) reaction, the fluidic path, including the feed lines, and discharge lines were rinsed with 8 ml toluene and 16 ml methanol, then dried with N_2_ flow. After changing reaction conditions, the flow reactor was allowed to stabilize for two residence times before a sample was collected by directing the selector valve towards a collection vial. Following the sample collection, the flow reactor effluent was directed to the waste collection vial during the transient period of the next reaction condition. Product analysis was performed by GC-MS and NMR.

### General Procedure 2: HAM in the batch reactor

The catalyst, dicarbonyl 2,4-pentanedionato rhodium(I) catalyst and the ligand, 4,6-Bis(diphenylphosphino)-10*H*-phenoxazine (N-xathphos), were dissolved in a stock solution of toluene and methanol at the target solvent composition under inert atmosphere inside a glovebox. The co-catalyst was weighed in an 8 ml glass vial and dissolved in a measured amount of the catalyst stock solution. The olefin and amine were subsequently added to the vial and the solution was further diluted with toluene/methanol to the desired concentration. The glass vial containing the liquid mixture and a magnetic stir bar was placed in an autoclave (Buchiglass Tinyclave, 35 mL under inert atmosphere). Supplementary Figure 3 shows a picture of the autoclave and the glass vial insert. The autoclave was connected to the gas supply manifold. Prior to each experiment, the lines and the autoclave were purged three times with N_2_. CO and H_2_ were then sequentially introduced into the autoclave at their target pressures. The autoclave was disconnected and placed in an oil bath heated with a temperature-controlled stir plate. The stirring rate was set at 800 rpm. A separate batch experiment performed at a stirring rate of 1100 rpm resulted in the same yield and selectivity obtained with the 800 rpm stirring rate reaction. Upon reaction completion, the autoclave was removed from the oil bath and cooled in a water bath until it reached room temperature. The autoclave was then vented through the manifold and purged three times with N_2_ before opening. Aliquots were taken from the reaction mixture. Product analysis was performed by GC-MS and NMR.

### Catalyst recycling procedure (Fig. 5)

Following General Procedure 2, 9.24 mg of the co-catalyst **2F** were weighed in an 8 ml glass vial. 1.5 ml of the toluene/methanol (1:4 volumetric raio) stock solution, containing 0.77 mg of the Rh catalyst and 6.6 mg of the ligand was transferred to the glass vial. 0.47 ml of 1-octene and 0.296 ml of piperidine were added and the solution was diluted to the desired concentration (1 M 1-octene) with toluene/methanol solvent. The reaction was performed at 115 °C for 60 min residence time after reaching thermal equilibrium. The cold pressure was 24 barg (total pressure) and the initial H_2_/CO ratio was 3.5. 50 μl aliquot was taken for analysis following the reaction completion and then the solvent was removed under high vacuum at 75 °C. The formed dispersion was redissolved in 2 ml pentane to precipitate the solid and the pentane was removed under high vacuum. The pentane wash was repeated three times. After the third time, the amine solution in pentane was separated from the solid by settling and transferred to another vial. The vial that contained the solid residue was transferred to a glovebox and redissolved in 1.5 ml toluene/methanol solvent. Next, fresh reactants were added as following: 0.47 ml of 1-octene, and 0.296 ml of piperidine, followed by dilution to the desired concentration (1 M 1-octene) by addition of toluene/methanol solvent. The vial was transferred to the autoclave for a new round of the HAM reaction with the recycled catalyst. In the experiment where fresh **2F** was added in every recycle, 4.6 mg of **2F** was weighed in the vial that contained the solid before adding the fresh solvent.

### On-the-fly switching from HAM to hydroaminovinylation (Fig. 6)

Following General Procedure 1, The solution for loading in the catalyst syringe was prepared by dissolving 5.16 mg (0.1 mol %) of the catalyst, dicarbonyl 2,4-pentanedionato rhodium(I), and 44.1 mg of the ligand, N-Xantphos, in 2 ml toluene (4 to 1 ligand to Rh ratio). The solution was diluted with 8 ml methanol solvent and loaded into an 8-ml stainless steel syringe. 1-octene (3.13 ml, 1 M) and piperidine (1.97 ml, 0.99 equiv.) were added to 61.6 mg of co-catalyst **2F** (2.0 mol %), diluted with toluene/methanol solvent to 10 ml total volume, and loaded into an 8 ml stainless steel syringe. The flow reactor pressure was set at 28 barg and the flow rate from the syringes was set at 8.48 μl/min each. The H_2_ and CO flow rates were set at 1.995 mln/min and 0.57 mln/min, respectively. The flow reactor temperature was set at 115 °C. The reaction was run for 40 min before collecting samples. The collected crude mixture was analyzed by GC-MS and the amine *l*/*b* was 84. To switch to condition (2), zero **2F** concentration, a third reactant syringe that contained 1-octene (3.13 ml, 1 M) and piperidine (1.97 ml, 0.99 equiv.) diluted with methanol solvent to 10 ml total was connected to the flow reactor and used as the reactant feed syringe. To switch to condition (3), the flow reactor temperature was lowered to 95 °C and the H_2_ and CO flow rates were set at 1.28 mln/min and 1.28 mln/min, respectively. To switch to condition (4), fresh catalyst and reactant syringes were prepared the same way as described for condition (1), except that methanol was replaced by toluene in both syringes and no co-catalyst **2F** was added. The flow reactor temperature was set at 115 °C and the H_2_ and CO flow rates were set at 1.71 mln/min and 0.86 mln/min, respectively. To switch to condition (5), a fresh reactant syringe was added that contained 61.6 mg of co-catalyst **2F** in toluene. To switch to condition (6), the flow reactor temperature was set at 125 °C. After switching to each new condition, the flow reactor was allowed to stabilize for 40 min before collecting samples for the shown length of time on Supplementary Fig. 1 (TOS). Samples were collected and analyzed at each condition and the amine and enamine yield are reported in Supplementary Fig. 1. The reaction was run under condition (6) for 4 h and the product was collected and solvent was removed under high vacuum. The residue was washed three times with pentane and the enamine product yield was measured at 72% with an *l*/*b* of 90, measured by GC-MS.

## Supplementary information

Supplementary Information

## Data Availability

The authors declare that all data supporting the findings of this study are available within the main text and Supplementary Information.
