# Supplementary material for: Recyclable cooperative catalyst for accelerated hydroaminomethylation of hindered amines in a continuous segmented flow reactor
Source: Nat Commun. 2022 May 4;13:2441. doi: 10.1038/s41467-022-30175-0 (PMC9068773; doi:10.1038/s41467-022-30175-0)
Supplement: Supplementary file 1 — Supplementary Information [file 41467_2022_30175_MOESM1_ESM.pdf]

Supplementary Information for

Recyclable Cooperative Catalyst for Accelerated Hydroaminomethylation of Hindered Amines in a Continuous Segmented Flow Reactor

Malek Y. S. Ibrahim[1], and Milad Abolhasani[1*]

[1]*Department of Chemical and Biomolecular Engineering, North Carolina State University; Raleigh, NC 27695 USA*

Correspondence to: abolhasani@ncsu.edu

1. Supplementary Methods 2

2. Supplementary Discussion 3

3. Supplementary References 41

1. Supplementary Methods

Materials

Reaction solvents, toluene (MilliporeSigma, 99.8% Anhydrous) and methanol (Supelco, ACS Grade) were degassed with argon and dried on molecular sieves before use. The olefin substrates, including 1-octene (Across, 99%), styrene (Across, 99%, stabilized), α-methylstyrene (Across, 99%, stabilized), and 1,5 hexadiene (Alfa Aesar, 98%) were degassed with argon and stored on molecular sieve under nitrogen before use. The amine reactants, including piperidine (Aldrich, 99.5%), morpholine (Oakwood, purified by distillation from glass), dihexylamine (Across, 99%), dicyclohexylamine (MilliporeSigma, 99%), 2-methylpiperidine (Across, 99%), 2-phenylpiperidine (TCI, 98%), 2,2,6,6-tetramethylpiperidine (Alfa Aesar 98%), cyclohexylamine (TCI, 99%), aniline (Thermo Fisher, 99+%), N-methylaniline (Thermo Fisher, 98%), and diphenylamine (Alfa Aesar, 98%) were degassed with argon and stored on molecular sieve under nitrogen before use. The catalyst, dicarbonyl 2,4-pentanedionato rhodium(I) (Alfa Aesar, 97%), and the ligands, including 4,5-Bis(diphenylphosphino)-9,9-dimethylxanthene, Xantphos (Strem, 98%), 4,6-Bis(diphenylphosphino)-10*H*-phenoxazine, 4,6- Bis(diphenylphosphino) phenoxazine, N-Xantphos (Alfa Aesar, 98%+), 9,9-Dimethyl-4,5-bis(di-*tert*-butylphosphino)xanthene, *t*-Bu-Xantphos (Aldrich, 97%), 2,2'-Bis(diphenylphosphinomethyl)-1,1'-biphenyl, BISBI (99%, Strem), and P,P′-(9,9-Dimethyl-9*H*-xanthene-4,5-diyl)bis[*N*,*N*,*N*′,*N*′-tetraethyl-phosphonous diamide] (MilliporeSigma, 97%) were used as received. The benzoic acids were purchased from Oakwood Chemical. 1,3,5-trimethoxybenzene (MilliporeSigma, 99%+) was used as the standard for gas chromatography (GC). 2-octene (mixture of cis and trans isomer) (TCI, 98%) and nonanal (Alfa Aesar, 95%) were used for GC calibration. Pentane solvent (Fisher, HPLC grade) was used for catalyst precipitation and wash during recycling. Deuterated solvents, including chloroform-d (Across, 99.8%) and chloroform-d (CIL, 99.8%), Toluene-d_8 (CIL, 99.5%), methanol-d_4 (CIL, 99.8%) were used to collect nuclear magnetic resonance (NMR) spectra. Carbon monoxide, hydrogen, argon, and nitrogen were purchased from Airgas at 99.9% purity. Deuterium (D_2) gas 99.96% D was purchased from MilliporeSigma.

Methods

50 µL of the reaction samples collected from either batch or flow reactor were diluted with 1 mL of toluene and 100 µL of 0.05 M 1,3,5-trimethoxybenzene in toluene as an internal calibration standard. 1 µL of the GC mixture was injected into Shimadzu GCMS-2010 with a Zebron ZB-5MSi column 30m × 0.25mm × 0.25µm.

GC method: 7 min at 40℃, followed by 20℃/min to 5 min at 85℃, and 20℃/min to 260℃. Component calibration was performed on 1-octene, cis-2-octene, trans-2-octene, n-octane, nonanal, 2-methyl octanal, and the purified amines and enamine products relative to the internal standard. The purity of isolated compounds was determined by GC.

All compounds were characterized by NMR and high-resolution mass spectrometry (HRMS). Linear to branched (*l*/*b*) ratios were calculated from GC-MS analysis of the crude mixture. NMR spectra were recorded on *Bruker NEO 400* (400 MHz) NMR spectrometer at room temperature.

HRMS analysis was carried out on a *Thermo Fisher Scientific Exactive Plus MS*, a benchtop full-scan Orbitrap™ mass spectrometer (70 ev), using Heated Electrospray Ionization (HESI). Samples were diluted in acetonitrile and analyzed via flow injection into the mass spectrometer at a flow rate of 200 µL/min. The mobile phase was 90% acetonitrile with 0.1% formic acid and 10% water with 0.1% formic acid. The mass spectrometer was operated in positive ion mode.

2. Supplementary Discussion

Ligand and Co-Catalyst Screening in flow HAM (Table 1)

Following **General Procedure 1**, the solution for loading in the catalyst syringe was prepared by dissolving 2.32 mg (0.1 mol %) of the catalyst, dicarbonyl 2,4-pentanedionato rhodium(I), and the corresponding ligand at 4 to 1 ligand to Rh ratio in 2 ml toluene separately: Xantphos, 20.83 mg; BISBI, 19.8 mg; N-Xantphos, 19.9 mg, *t*-Bu-Xantphos, 18 mg, and P,P′-(9,9-Dimethyl-9*H*-xanthene-4,5-diyl)bis[*N*,*N*,*N*′,*N*′-tetraethyl-phosphonous diamide], 20.1 mg. The catalyst solution was then diluted with 8 ml methanol and loaded into an 8-ml stainless steel syringe. 1-octene (1.41 ml, 0.45 M) and piperidine (0.89 ml, 1.01 equiv.) were added to **7** mg of the co-catalyst **2F** (0.5 mol %) and diluted with toluene/methanol solvent to 10 ml total volume. The prepared solution of the olefin, amine, and co-catalyst was then loaded an 8-ml stainless steel syringe. The flow reactor pressure was set at 24 barg and the flow rate from each syringe was set at 13.73 μl/min. The H_2 and CO flow rates were set at 1.855 mln/min and 0.53 mln/min, respectively. The flow reactor temperature was set at 125°C. The reaction was run for 30 min before collecting samples for another 30 min at each entry for analysis by GC-MS. Different acids were tested in flow and the results are presented in **Supplementary Table 1**.

HAM Reaction Condition Optimization (Fig. 3)

Optimization of 2F loading. Following **General Procedure 1**, the solution for loading in the catalyst syringe was prepared by dissolving 2.32 mg (0.1 mol %) of the catalyst, dicarbonyl 2,4-pentanedionato rhodium(I), and 19.9 mg of the ligand, N-Xantphos, in 2 ml toluene (4 to 1 ligand to Rh ratio). The solution was then diluted with 8 ml methanol and loaded into an 8-ml stainless steel syringe. 1-octene (1.41 ml, 0.45 M) and piperidine (0.89 ml, 1.01 equiv.) were added to 27.8 mg of the co-catalyst **2F** (2.0 mol %), diluted with toluene/methanol solvent to 10 ml total volume, and loaded into an 8-ml stainless steel syringe. Another solution was prepared the same, but without the addition of **2F** and loaded into an 8-ml stainless steel syringe. The flow reactor pressure was set at 24 barg and the flow rate from the catalyst syringe was set at 13.73 μl/min. The total flow rate from the two reactant syringes was also maintained at 13.73 μl/min but the volumetric ratio of the reactant syringes was varied to vary the **2F** loading in the flow reactor. The H_2 and CO flow rates were set at 1.855 mln/min and 0.53 mln/min, respectively. The flow reactor temperature was set at 125°C. The reaction was run for 30 min before collecting samples for another 30 min at each entry for analysis by GC-MS.

Supplementary Table 1. *Effect of the co-catalyst structure on in-flow HAM with Rh/N-Xantphos catalyst*.

Co-catalyst	Amine Yield (%)	Amine/Enamine Ratio	Amine *l*/*b*
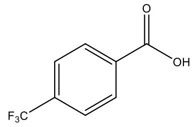	71.8	15.8	54.4
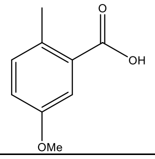	73	14.7	61.4
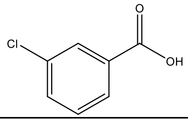	73.4	21.8	60.3
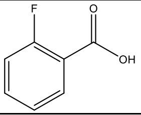	71.2	14.3	76.9
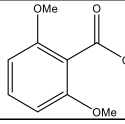	74	24.7	65.3
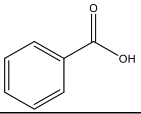	74.3	20.8	59.4
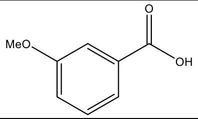	75.7	25.6	72.9
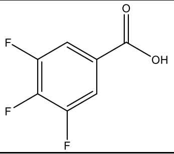	75.1	35.6	53.4
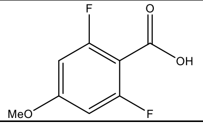	76.8	27.2	71.5
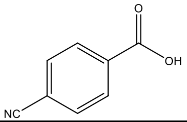	78.2	48.5	57.8
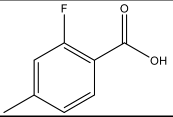	80	36.6	74.3

Optimization of solvent composition. Following **General Procedure 1**, the solution for loading in the catalyst syringe was prepared by dissolving 2.32 mg (0.1 mol %) of the catalyst, dicarbonyl 2,4-pentanedionato rhodium(I), and 19.9 mg of the ligand, N-Xantphos, in 2 ml toluene (4 to 1 ligand to Rh ratio). The solution was then diluted with 8 ml methanol solvent and loaded into an 8-ml stainless steel syringe. Another catalyst solution syringe was prepared the same way but diluted with toluene instead of methanol. 1-octene (1.41 ml, 0.45 M) and piperidine (0.89 ml, 1.01 equiv.) were added to 27.8 mg of the co-catalyst **2F** (2.0 mol %), diluted with toluene solvent at the target solvent ratio to 10 ml total volume and, and loaded into an 8-ml stainless steel syringe. Another reactant syringe was prepared the same way but diluted with methanol instead of toluene. The two reactant syringes were connected to T-junction (IDEX Health & Sciences) that was connected to the feed stream mixing cross junction (IDEX Health & Sciences) with Teflon tubing (1/16" OD FEP). The flow reactor pressure was set at 24 barg and the flow rate from the two catalyst syringes and the two reactant syringes was set at 13.73 μl/min for each but the volumetric ratio was varied to match the desired solvent composition at constant catalyst and reactant concentrations. The H_2 and CO flow rates were set at 1.855 mln/min and 0.53 mln/min, respectively. The flow reactor temperature was set at 125°C. The reaction was run for 30 min before collecting samples for another 30 min at each entry for analysis by GC-MS.

Optimization of H_2/CO ratio. Following **General Procedure 1**, the solution for loading in the catalyst syringe was prepared by dissolving 2.32 mg (0.1 mol %) of the catalyst, dicarbonyl 2,4-pentanedionato rhodium(I), and 19.9 mg of the ligand, N-Xantphos, in 2 ml toluene (4 to 1 ligand to Rh ratio). The solution was diluted with 8 ml methanol solvent and loaded into an 8-ml stainless steel syringe. 1-octene (1.41 ml, 0.45 M) and piperidine (0.89 ml, 1.01 equiv.) were added to **27.8** mg of the co-catalyst **2F** (2.0 mol %), diluted with methanol solvent to 10 ml total volume, and loaded into an 8-ml stainless steel syringe. The flow reactor pressure was set at 24 barg and the flow rate from the syringes was set at 13.73 μl/min each. The H_2 and CO flow rates were varied to achieve variable H_2/CO ratio, while maintaining the total gas flow rate constant at 2.385 mln/min. The flow reactor temperature was set at 125°C. The reaction was run for 30 min before collecting samples for another 30 min at each entry for analysis by GC-MS.

Product Characterization

1-Nonylpiperidine: 1a

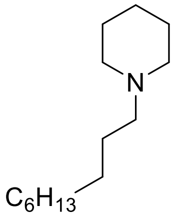

Following **General Procedure 1**, the solution for loading in the catalyst syringe was prepared by dissolving 5.16 mg (0.1 mol %) of dicarbonyl 2,4-pentanedionato rhodium(I) catalyst and 44.1 mg of N-Xantphos ligand in 2 ml toluene. The solution was diluted with 8 ml methanol solvent and loaded into an 8-ml stainless steel syringe. 1-octene (3.13 ml, 1 M) and piperidine (1.97 ml, 0.99 equiv.) were added to 61.6 mg of the co-catalyst **2F** (2.0 mol %), diluted with 1:4 toluene:methanol solvent ratio to 10 ml total volume, and loaded into an 8-ml stainless steel syringe. The flow reactor pressure was set at 28 barg and the flow rate from the syringes was set at 8.48 μl/min each. The H_2 and CO flow rates were set at 1.995 mln/min and 0.57 mln/min, respectively. The flow reactor temperature was set at 115°C. The reaction was run for 40 min before collecting samples for another 4 h. The collected crude mixture was analyzed by GC-MS and the amine *l*/*b* was 84. The solvent was removed under high vacuum at 75°C and the liquid residue was dissolved in pentane and separated from the solid precipitate in a separate vial. Next, pentane was removed under high vacuum. The product was isolated as colorless liquid with a mass of 776 mg (92% yield).[1]

^{1}H NMR (400 MHz, $CDCl_3$) δ 2.35 (s, 4H), 2.33 – 2.21 (m, 2H), 1.57 (p, *J* = 5.7 Hz, 4H), 1.51 – 1.35 (m, 4H), 1.33 – 1.18 (m, 12H), 0.85 (t, *J* = 6.8 Hz, 3H).
HRMS: calcd for $[C_{14}H_{29}NH]^+$ 212.23728; found 212.23708

4-Nonylmorpholine: 1b

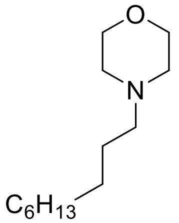

Following **General Procedure 1**, the solution for loading in the catalyst syringe was prepared by dissolving 5.16 mg (0.1 mol %) of dicarbonyl 2,4-pentanedionato rhodium(I) catalyst and 44.1 mg of N-Xantphos ligand in 2 ml toluene. The solution was diluted with 8 ml methanol solvent and loaded into an 8-ml stainless steel syringe. 1-octene (3.13 ml, 1 M) and morpholine (1.75 ml, 0.99 equiv.) were added to 61.6 mg of the co-catalyst **2F** (2.0 mol %), diluted with 1:4 toluene:methanol

solvent ratio to 10 ml total volume, and loaded into an 8-ml stainless steel syringe. The flow reactor pressure was set at 28 barg and the flow rate from the syringes was set at 8.48 μl/min each. The H_2 and CO flow rates were set at 1.995 mln/min and 0.57 mln/min, respectively. The flow reactor temperature was set at 115°C. The reaction was run for 40 min before collecting samples for another 4 h. The collected crude mixture was analyzed by GC-MS and the amine *l*/*b* was 56. The solvent was removed under high vacuum at 75 °C and the liquid residue was dissolved in pentane and separated from the solid precipitate in a separate vial. Next, pentane was removed under high vacuum. The product was isolated as colorless liquid with a mass of 738 mg (86.6% yield). [1]
^{1}H NMR (400 MHz, $CDCl_3$) δ 3.77 (t, *J* = 4.7 Hz, 4H), 2.52 (t, *J* = 4.7 Hz, 4H), 2.39 (p, *J* = 7.1 Hz, 2H), 1.61 – 1.47 (m, 2H), 1.35 – 1.18 (m, 12H), 0.87 (t, *J* = 6.8 Hz, 3H).
HRMS: calcd for $[C_{13}H_{28}NO]^+$ 214.21654; found 214.21631

***N,N*-dihexylnonan-1-amine: 1c**

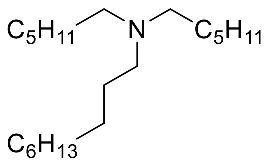

Following **General Procedure 1**, the solution for loading in the catalyst syringe was prepared by dissolving 5.16 mg (0.1 mol %) of dicarbonyl 2,4-pentanedionato rhodium(I) catalyst and 44.1 mg of N-Xantphos ligand in 2 ml toluene. The solution was diluted with 8 ml methanol solvent and loaded into an 8-ml stainless steel syringe. 1-octene (3.13 ml, 1 M) and dihexylamine (4.66 ml, 0.99 equiv.) were added to 61.6 mg **2F** (2.0 mol %) and diluted with 1:4 toluene:methanol solvent ratio to 10 ml total volume and one reactant syringe was filled with the solution. The flow reactor pressure was set at 28 barg and the flow rate from the syringes was set at 5.2 μl/min each. The H_2 and CO flow rates were set at 1.225 mln/min and 0.35 mln/min, respectively. The flow reactor temperature was set at 115 °C. The reaction was run for 60 min before collecting samples for another 6.4 h. The collected crude mixture was analyzed by GC-MS and the amine *l*/*b* was 198. The solvent was removed under high vacuum at 75 °C and the liquid residue was dissolved in pentane and separated from the solid precipitate in a separate vial. Next, pentane was removed under high vacuum. The product was isolated as colorless liquid with mass of a 1039 mg (83.6% yield). [1]

^{1}H NMR (400 MHz, $CDCl_3$) δ 2.40 (dd, *J* = 8.9, 6.4 Hz, 6H), 1.42 (ddd, *J* = 14.8, 7.2, 4.3 Hz, 6H), 1.37 – 1.19 (m, 24H), 0.88 (td, *J* = 6.9, 1.9 Hz, 9H).
HRMS: calcd for $[C_{21}H_{46}N]^+$ 312.36248; found 312.36235

N-methyl-N-nonylaniline: 1d

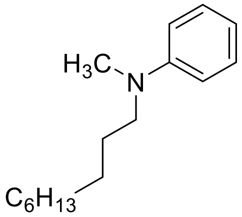

Following **General Procedure 1**, the solution for loading in the catalyst syringe was prepared by dissolving 5.16 mg (0.1 mol %) of dicarbonyl 2,4-pentanedionato rhodium(I) catalyst and 44.1 mg of N-Xantphos ligand in 2 ml toluene. The solution was diluted with 8 ml methanol solvent and loaded into an 8-ml stainless steel syringe. 1-octene (3.13 ml, 1 M) and N-methylaniline (2.17 ml, 0.99 equiv.) were added to 61.6 mg co-catalyst **2F** (2.0 mol %) and diluted with 1:4 toluene:methanol solvent ratio to 10 ml total volume and one reactant syringe was filled with the solution. The flow reactor pressure was set at 28 barg and the flow rate from the syringes was set at 6.24 μl/min each. The H_2 and CO flow rates were set at 1.47 mln/min and 0.42 mln/min, respectively. The flow reactor temperature was set at 115 ^oC. The reaction was run for 60 min before collecting samples for another 5.3 h. The collected crude mixture was analyzed by GC-MS and the amine *l*/*b* was 159. The solvent was removed under high vacuum at 75 ^oC and the liquid residue was dissolved in pentane and separated from the solid precipitate in a separate vial. Next, pentane was removed under high vacuum. The product was isolated as colorless liquid with a mass of 801 mg (86% yield).[2]

^1H NMR (400 MHz, $CDCl_3$) δ 7.27 – 7.16 (m, 2H), 6.74 – 6.63 (m, 3H), 3.36 – 3.25 (m, 2H), 2.92 (s, 3H), 1.57 (dd, *J* = 10.9, 4.0 Hz, 2H), 1.37 – 1.22 (m, 12H), 0.93 – 0.85 (m, 3H).

HRMS: calcd for $[C_{16}H_{28}N]^+$ 234.22163; found 234.22147

2-methyl-N-nonylpiperidine: 1e

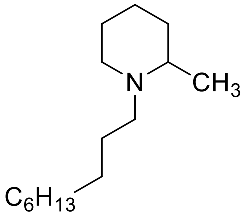

Following **General Procedure 1**, the solution for loading in the catalyst syringe was prepared by dissolving 5.16 mg (0.1 mol %) of dicarbonyl 2,4-pentanedionato rhodium(I) catalyst and 44.1 mg of N-Xantphos ligand in 2 ml toluene. The solution was diluted with 8 ml methanol solvent and loaded into an 8-ml stainless steel syringe. 1-octene (3.13 ml, 1 M) and 2-methylpiperidine (2.37 ml, 0.99 equiv.) were added to 154 mg co-catalyst **2F** (5.0 mol %) and diluted with 1:4 toluene:methanol solvent ratio to 10 ml total volume and one reactant syringe was filled with the solution. The flow reactor pressure was set at 28 bar and the flow rate from the syringes was set at

8.48 μl/min each. The H_2 and CO flow rates were set at 1.995 mln/min and 0.57 mln/min, respectively. The flow reactor temperature was set at 115 °C. The reaction was run for 40 min before collecting samples for another 4 h. The collected crude mixture was analyzed by GC-MS and the amine *l*/*b* was 213. The solvent was removed under high vacuum at 75 °C and the liquid residue was dissolved in pentane and separated from the solid precipitate in a separate vial. Next, pentane was removed under high vacuum. The product was isolated as colorless liquid with a mass of 711 mg (79% yield).

^{1}H NMR (400 MHz, $CDCl_3$) δ 2.86 (dd, *J* = 9.8, 5.6 Hz, 1H), 2.66 (ddd, *J* = 13.0, 10.2, 6.0 Hz, 1H), 2.41 – 2.26 (m, 2H), 2.16 (dt, *J* = 10.9, 5.3 Hz, 1H), 1.72 – 1.40 (m, 6H), 1.41 – 1.17 (m, 14H), 1.07 (d, *J* = 6.3 Hz, 3H), 0.93 – 0.81 (m, 3H).

^{13}C NMR (101 MHz, $CDCl_3$) δ 55.82, 54.18, 52.17, 34.60, 31.90, 29.63 (d, *J* = 4.1 Hz), 29.30, 27.86, 26.15, 25.09, 24.08, 22.68, 14.11.

HRMS: calcd for $[C_{15}H_{32}N]^+$ 226.25293; found 226.25253

1-nonyl-2-phenylpiperidine: 1f

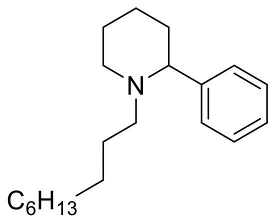

Following **General Procedure 1**, the solution for loading in the catalyst syringe was prepared by dissolving 5.16 mg (0.1 mol %) of dicarbonyl 2,4-pentanedionato rhodium(I) catalyst and 44.1 mg of N-Xantphos ligand in 2 ml toluene. The solution was diluted with 8 ml methanol solvent and loaded into an 8-ml stainless steel syringe. 1-octene (3.13 ml, 1 M) and 2-phenylpiperidine (4.03 ml, 0.99 equiv.) were added to 154 mg co-catalyst **2F** (5.0 mol %) and diluted with 1:4 toluene:methanol solvent ratio to 10 ml total volume and one reactant syringe was filled with the solution. The flow reactor pressure was set at 28 barg and the flow rate from the syringes was set at 8.48 μl/min each. The H_2 and CO flow rates were set at 1.995 mln/min and 0.57 mln/min, respectively. The flow reactor temperature was set at 115 °C. The reaction was run for 40 min before collecting samples for another 4 h. The collected crude mixture was analyzed by GC-MS and the amine l/b was 87. The solvent was removed under high vacuum at 75 °C and the liquid residue was dissolved in pentane and separated from the solid precipitate in a separate vial. Next, pentane was removed under high vacuum. The product was isolated as colorless liquid with a mass of 1022 mg (89% yield).

^{1}H NMR (400 MHz, $CDCl_3$) δ 7.51 – 7.06 (m, 5H), 3.21 (d, *J* = 11.6 Hz, 1H), 3.03 (dd, *J* = 11.0, 3.0 Hz, 1H), 2.44 (dt, *J* = 12.7, 8.1 Hz, 1H), 2.17 – 1.97 (m, 1H), 1.96 – 1.83 (m, 1H), 1.81 – 1.48 (m, 4H), 1.44 – 0.96 (m, 13H), 0.86 (t, *J* = 7.0 Hz, 3H).

^{13}C NMR (101 MHz, $CDCl_3$) δ 145.38, 131.72 – 117.79 (m), 68.52, 56.68, 36.69 (t, J = 126.7 Hz), 32.29 – 19.85 (m), 15.37 (d, J = 124.6 Hz), 12.27
HRMS: calcd for $[C_{20}H_{34}N]^+$ 288.26858; found 288.26838

_N,N_-dicylohexyl-_N_-nonylamine: 1g

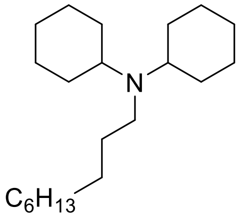

Following **General Procedure 1**, the solution for loading in the catalyst syringe was prepared by dissolving 5.16 mg (0.1 mol %) of dicarbonyl 2,4-pentanedionato rhodium(I) catalyst and 44.1 mg of N-Xantphos ligand in 2 ml toluene. The solution was diluted with 8 ml methanol solvent and loaded into an 8-ml stainless steel syringe. 1-octene (3.13 ml, 1 M) and dicyclohexylamine (4.0 ml, 0.99 equiv.) were added to 308 mg co-catalyst **2F** (10.0 mol %) and diluted with 1:4 toluene:methanol solvent ratio to 10 ml total volume and one reactant syringe was filled with the solution. The flow reactor pressure was set at 28 barg and the flow rate from the syringes was set at 1.19 μl/min each. The H_2 and CO flow rates were set at 0.28 mln/min and 0.08 mln/min, respectively. The flow reactor temperature was set at 115 °C. The reaction was run for 260 min before collecting samples for another 7 h. The collected crude mixture was analyzed by GC-MS and the amine l/b was 58. The solvent was removed under high vacuum at 75 °C and the liquid residue was dissolved in pentane and separated from the solid precipitate in a separate vial. Next, pentane was removed under high vacuum. The product was isolated as white crystals with a mass of 218 mg (71% yield).
^{1}H NMR (400 MHz, $CDCl_3$) δ 2.70 – 2.32 (m, 4H), 1.74 (s, 8H), 1.64 – 1.56 (m, 3H), 1.41 – 1.03 (m, 23H), 0.93 – 0.80 (m, 3H).
^{13}C NMR (101 MHz, $CDCl_3$) δ 58.13, 31.92, 31.67, 29.67 (d, J = 6.2 Hz), 29.32, 27.46, 26.42, 22.69, 14.12.
HRMS: calcd for $[C_{21}H_{42}N]^+$ 308.33118; found 308.33120

2,2,6,6-tetramethyl-1-nonylpiperidine: 1h

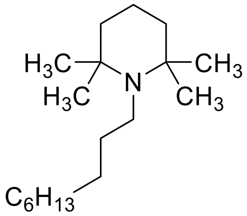

Following **General Procedure 1**, the solution for loading in the catalyst syringe was prepared by dissolving 5.16 mg (0.1 mol %) of dicarbonyl 2,4-pentanedionato rhodium(I) catalyst and 44.1 mg of N-Xantphos ligand in 2 ml toluene. The solution was diluted with 8 ml methanol solvent and loaded into an 8-ml stainless steel syringe. 1-octene (3.13 ml, 1 M) and 2,2,6,6-tetramethylpiperidine (3.21 ml, 0.99 equiv.) were added to 308 mg co-catalyst **2F** (10.0 mol %) and diluted with 1:4 toluene:methanol solvent ratio to 10 ml total volume and one reactant syringe was filled with the solution. The flow reactor pressure was set at 28 barg and the flow rate from the syringes was set at 1.19 μl/min each. The H_2 and CO flow rates were set at 0.28 mln/min and 0.08 mln/min, respectively. The flow reactor temperature was set at 115°C. The reaction was run for 260 min before collecting samples for another 7 h. The collected crude mixture was analyzed by GC-MS and no product was formed.

_N_-nonyl-_N_-phenylaniline: 1i

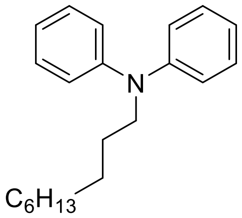

Following **General Procedure 1**, the solution for loading in the catalyst syringe was prepared by dissolving 5.16 mg (0.1 mol %) of dicarbonyl 2,4-pentanedionato rhodium(I) catalyst and 44.1 mg of N-Xantphos ligand in 2 ml toluene. The solution was diluted with 8 ml methanol solvent and loaded into an 8-ml stainless steel syringe. 1-octene (3.13 ml, 1 M) and diphenylamine (3.418 g, 0.99 equiv.) were added to 308 mg co-catalyst **2F** (10.0 mol %) and diluted with 1:4 toluene:methanol solvent ratio to 10 ml total volume and one reactant syringe was filled with the solution. The flow reactor pressure was set at 28 barg and the flow rate from the syringes was set at 1.19 μl/min each. The H_2 and CO flow rates were set at 0.28 mln/min and 0.08 mln/min respectively. The flow reactor temperature was set at 115 °C. The reaction was run for 260 min before collecting samples for another 7 h. The collected crude mixture was analyzed by GC-MS and the amine *l*/*b* was 120. The solvent was removed under high vacuum at 75 °C and the liquid residue was dissolved in pentane and separated from the solid precipitate in a separate vial. Next, pentane was removed under high vacuum. The product was isolated as yellow crystals with a mass of 224 mg (76% yield).

^{1}H NMR (400 MHz, $CDCl_3$) δ 7.31 – 7.21 (m, 2H), 7.11 – 7.04 (m, 2H), 7.02 – 6.89 (m, 6H), 3.76 – 3.54 (m, 2H), 1.66 (t, *J* = 7.5 Hz, 2H), 1.27 (d, *J* = 16.8 Hz, 12H), 0.88 (t, *J* = 6.7 Hz, 3H).
^{13}C NMR (101 MHz, $CDCl_3$) δ 148.13, 143.13, 129.29 (d, *J* = 12.5 Hz), 120.97 (d, *J* = 11.7 Hz), 117.82, 52.38, 31.87, 30.25 – 28.30 (m), 27.28 (d, *J* = 35.3 Hz), 22.67, 14.12, 0.01.
HRMS: calcd for $[C_{21}H_{30}N]^+$ 296.23728; found 296.23738

1-(3-phenylpropyl)piperidine: 1j

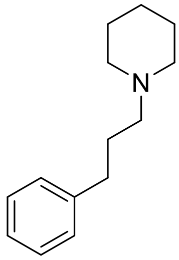

L+B

Following **General Procedure 1**, the solution for loading in the catalyst syringe was prepared by dissolving 5.16 mg (0.1 mol %) of dicarbonyl 2,4-pentanedionato rhodium(I) catalyst and 44.1 mg of N-Xantphos ligand in 2 ml toluene. The solution was diluted with 8 ml methanol solvent and loaded into an 8-ml stainless steel syringe. Styrene (2.29 ml, 1 M) and piperidine (1.98 ml, 0.99 equiv.) were added to 61.6 mg co-catalyst **2F** (2.0 mol %) and diluted with 1:4 toluene:methanol solvent ratio to 10 ml total volume and one reactant syringe was filled with the solution. The flow reactor pressure was set at 28 barg and the flow rate from the syringes was set at 5.2 μl/min each. The H_2 and CO flow rates were set at 1.225 mln/min and 0.35 mln/min, respectively. The flow reactor temperature was set at 115°C. The reaction was run for 60 min before collecting samples for another 6.4 h. The collected crude mixture was analyzed by GC-MS and the amine l/b was 2.1. The solvent was removed under high vacuum at 75 ^{o}C and the liquid residue was dissolved in pentane and separated from the solid precipitate in a separate vial. Next, pentane was removed under high vacuum. The product was isolated as colorless liquid with a mass of 658 mg (81% yield). [1]

^{1}H NMR (400 MHz, $CDCl_3$) δ 7.28 – 7.05 (m, 13.1H), 2.94 (q, *J* = 7.1 Hz, 1H), 2.59 – 2.50 (m, 3H), 2.40 – 2.16 (m, 14.3H), 1.83 (tt, *J* = 10.1, 7.0, 5.6 Hz, 3.32H), 1.56 (ddt, *J* = 11.7, 6.0 Hz, 9.85H), 1.42 (dp, *J* = 11.7, 6.0 Hz, 4.78H), 1.27 (d, *J* = 6.9 Hz, 3H).
HRMS: calcd for $[C_{14}H_{22}N]^+$ 204.17468; found 204.17462

1-(3-phenylbutyl)piperidine: 1k

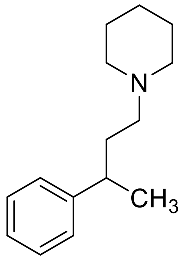

Following **General Procedure 1**, the solution for loading in the catalyst syringe was prepared by dissolving 5.16 mg (0.1 mol %) of dicarbonyl 2,4-pentanedionato rhodium(I) catalyst and 44.1 mg of N-Xantphos ligand in 2 ml toluene. The solution was diluted with 8 ml methanol solvent and

loaded into an 8-ml stainless steel syringe. Alpha-methylstyrene (2.59 ml, 1 M) and piperidine (1.98 ml, 0.99 equiv.) were added to 61.6 mg co-catalyst **2F** (2.0 mol %) and diluted with 1:4 toluene:methanol solvent ratio to 10 ml total volume and one reactant syringe was filled with the solution. The flow reactor pressure was set at 28 barg and the flow rate from the syringes was set at 1.29 μl/min each. The H_2 and CO flow rates were set at 0.305 mln/min and 0.087 mln/min, respectively. The reactor temperature was set at 135^oC. The reaction was run for 240 min before collecting samples for another 6.4 h. The collected crude mixture was analyzed by GC-MS and the amine *l*/*b* was 66. The solvent was removed under high vacuum at 75 ^oC and the liquid residue was dissolved in pentane and separated from the solid precipitate in a separate vial. Next, pentane was removed under high vacuum. The product was isolated as colorless liquid with a mass of 161 mg (74% yield).[3]

^1H NMR (400 MHz, $CDCl_3$) δ 7.20 (d, *J* = 7.2 Hz, 2H), 7.11 (dd, *J* = 7.4, 1.2 Hz, 3H), 2.63 (h, *J* = 7.0 Hz, 1H), 2.33 – 2.15 (m, 4H), 2.07 (ddd, *J* = 12.1, 9.9, 5.7 Hz, 2H), 1.81 – 1.61 (m, 2H), 1.49 (p, *J* = 5.6 Hz, 4H), 1.40 – 1.26 (m, 2H), 1.18 (d, *J* = 7.0 Hz, 3H).
HRMS: calcd for $[C_{15}H_{24}N]^+$ 218.19033; found 218.19021

***N*-nonylaniline: 1l**

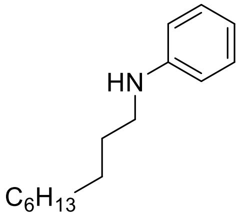

Following **General Procedure 1**, the solution for loading in the catalyst syringe was prepared by dissolving 5.16 mg (0.1 mol %) of dicarbonyl 2,4-pentanedionato rhodium(I) catalyst and 44.1 mg of N-Xantphos ligand in 2 ml toluene. The solution was diluted with 8 ml methanol solvent and loaded into an 8-ml stainless steel syringe. 1-octene (3.13 ml, 1 M) and aniline (1.84 ml, 0.99 equiv.) were added to 61.6 mg co-catalyst **2F** (2.0 mol %) and diluted with 1:4 toluene:methanol solvent ratio to 10 ml total volume and one reactant syringe was filled with the solution. The flow reactor pressure was set at 28 barg and the flow rate from the syringes was set at 5.2 μl/min each. The H_2 and CO flow rates were set at 1.225 mln/min and 0.35 mln/min, respectively. The flow reactor temperature was set at 115^oC. The reaction was run for 60 min before collecting samples for another 6.4 h. The collected crude mixture was analyzed by GC-MS and the amine *l*/*b* was 35. The solvent was removed under high vacuum at 75^oC and the liquid residue was dissolved in pentane and separated from the solid precipitate in a separate vial. Next, pentane was removed under high vacuum. The product was isolated as colorless liquid with a mass of 675 mg (77% yield).[1]

^{1}H NMR (400 MHz, $CDCl_3$) δ 7.24 – 7.12 (m, 2H), 6.75 – 6.65 (m, 1H), 6.65 – 6.56 (m, 2H), 3.10 (t, *J* = 7.1 Hz, 2H), 1.62 (p, *J* = 7.1 Hz, 2H), 1.37 – 1.22 (m, 12H), 0.93 – 0.83 (m, 3H).
HRMS: calcd for $[C_{15}H_{26}N]^+$ 220.20598; found 220.20579

1-Nonylcyclohexyalamine 1m

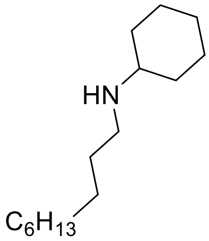

Following **General Procedure 1**, the solution for loading in the catalyst syringe was prepared by dissolving 5.16 mg (0.1 mol %) of dicarbonyl 2,4-pentanedionato rhodium(I) catalyst and 44.1 mg of N-Xantphos ligand in 2 ml toluene. The solution was diluted with 8 ml methanol solvent and loaded into an 8-ml stainless steel syringe. 1-octene (3.13 ml, 1 M) and cyclohexylamine (2.17 ml, 0.99 equiv.) were diluted with 1:4 toluene:methanol solvent ratio to 10 ml total volume and one reactant syringe was filled with the solution. The flow reactor pressure was set at 28 barg and the flow rate from the syringes was set at 8.48 μl/min each. The H_2 and CO flow rates were set at 1.71 and 0.855 mln/min respectively. The reactor temperature was set at 115 ^oC. The reaction was run for 40 min before collecting samples for another 8 h. Six ml of the collected liquid was added to 18.5 mg co-catalyst **2F** (2.0 mol %) and fed to a syringe for the second step. The reactor pressure was set at 25 bar and the flow rate from the syringe was set at 15.28 μl/min. The H_2 and CO flow rates were set at 2.0 mln/min and zero mln/min, respectively. The flow reactor temperature was set at 115^oC. The reaction was run for 40 min before collecting samples for another 4 h. The collected crude mixture was analyzed by GC-MS and the amine *l*/*b* was 62. The solvent was removed under high vacuum at 75 ^oC and the liquid residue was dissolved in pentane and separated from the solid precipitate in a separate vial. Next, pentane was removed under high vacuum. The product was isolated as colorless liquid with a mass of 711 mg (79% yield).[1]
^{1}H NMR (400 MHz, $CDCl_3$) δ 2.65 – 2.57 (m, 1H), 2.41 (tt, *J* = 10.5, 3.8 Hz, 1H), 1.94 – 1.83 (m, 2H), 1.72 (dt, *J* = 12.9, 3.6 Hz, 2H), 1.66 – 1.53 (m, 1H), 1.47 (p, *J* = 7.2 Hz, 2H), 1.35 – 1.19 (m, 10H), 1.17 – 0.99 (m, 2H), 0.88 (t, *J* = 6.7 Hz, 3H).
HRMS: calcd for $[C_{15}H_{32}N]^+$ 226.25293; found 226.25266

On-the-Fly Switching from HAM to Hydroaminovinylation (Fig. 6)

Following **General Procedure 1**, The solution for loading in the catalyst syringe was prepared by dissolving 5.16 mg (0.1 mol %) of the catalyst, dicarbonyl 2,4-pentanedionato rhodium(I) and 44.1 mg of the ligand, N-Xantphos, in 2 ml toluene (4 to 1 ligand to Rh ratio). The solution was diluted with 8 ml methanol solvent and loaded into an 8-ml stainless steel syringe. 1-octene (3.13 ml, 1 M) and piperidine (1.97 ml, 0.99 equiv.) were added to 61.6 mg of co-catalyst **2F** (2.0 mol %), diluted with toluene/methanol solvent to 10 ml total volume, and loaded into an 8-ml stainless steel syringe. The flow reactor pressure was set at 28 barg and the flow rate from the syringes was set at 8.48 μl/min each. The H_2 and CO flow rates were set at 1.995 mln/min and 0.57 mln/min, respectively. The flow reactor temperature was set at 115°C. The reaction was run for 40 min before collecting samples. The collected crude mixture was analyzed by GC-MS and the amine *l*/*b* was 84. To switch to condition (2), zero **2F** concentration, a third reactant syringe that contained 1-octene (3.13 ml, 1 M) and piperidine (1.97 ml, 0.99 equiv.) diluted with methanol solvent to 10 ml total was connected to the flow reactor and used as the reactant feed syringe. To switch to condition (3), the flow reactor temperature was lowered to 95°C and the H_2 and CO flow rates were set at 1.28 mln/min and 1.28 mln/min, respectively. To switch to condition (4), fresh catalyst and reactant syringes were prepared the same way as described for condition (1), except that methanol was replaced by toluene in both syringes and no co-catalyst **2F** was added. The flow reactor temperature was set at 115 °C and the H_2 and CO flow rates were set at 1.71 mln/min and 0.86 mln/min, respectively. To switch to condition (5), a fresh reactant syringe was added that contained 61.6 mg of co-catalyst **2F** in toluene. To switch to condition (6), the flow reactor temperature was set at 125°C. After switching to each new condition, the flow reactor was allowed to stabilize for 40 min before collecting samples for the shown length of time on **Supplementary Fig. 1** (TOS). Samples were collected and analyzed at each condition and the amine and enamine yield are reported in **Supplementary Fig. 1**. The reaction was run under condition (6) for 4 h and the product was collected and solvent was removed under high vacuum. The residue was washed three times with pentane and the enamine product yield was measured at 72% with an *l*/*b* of 90, measured by GC-MS.

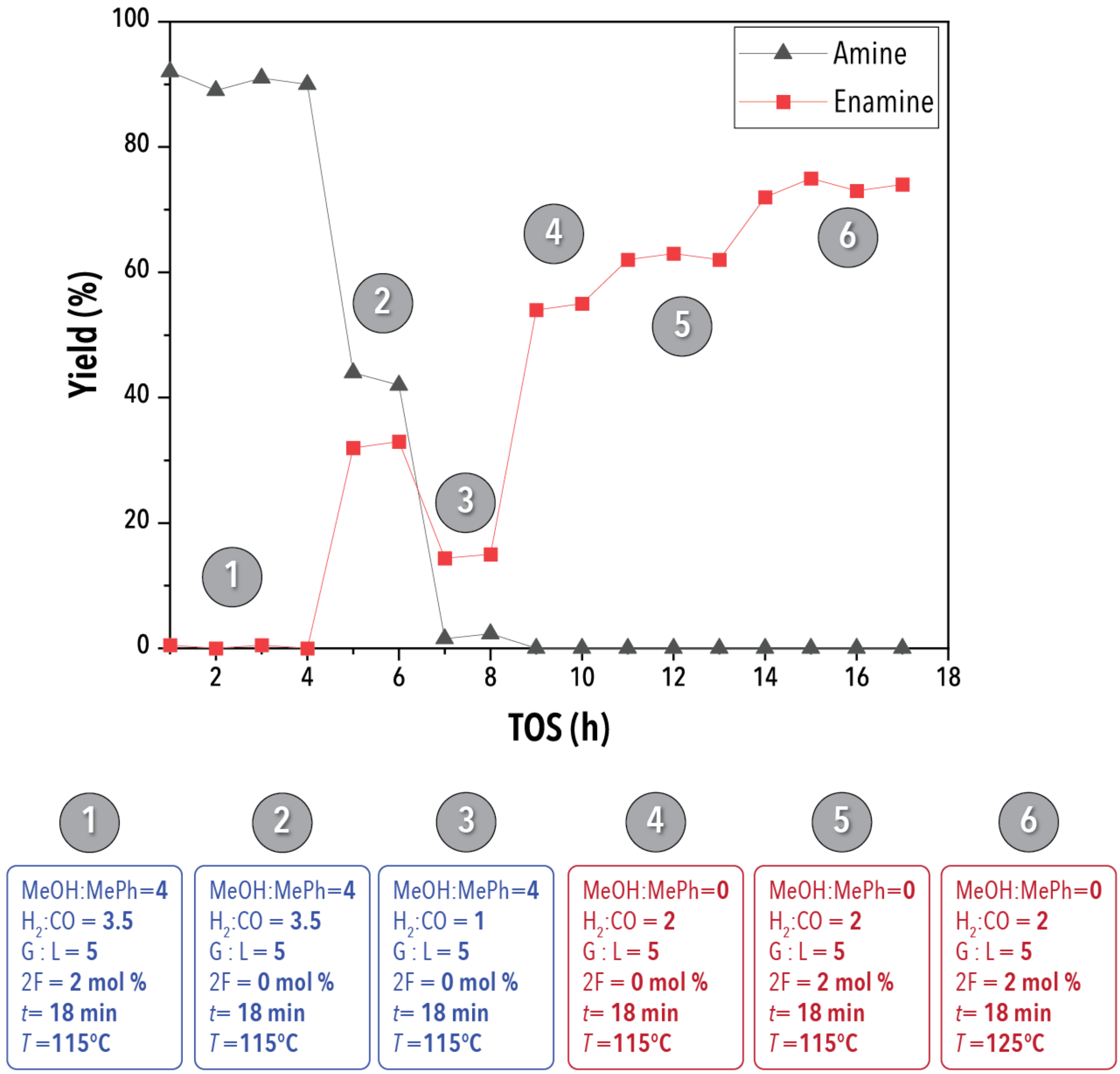

Supplementary Fig. 1. *On-the-fly switching from HAM to Hydroaminovinylation.*

Characterization of Hydroaminovinylation product 2a

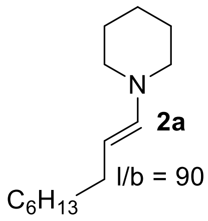

^{1}H NMR (400 MHz, $CDCl_3$) δ 5.81 (d, *J* = 13.8 Hz, 1H), 4.39 (dt, *J* = 13.9, 7.0 Hz, 1H), 2.76 – 2.69 (m, 4H), 1.94 (q, *J* = 6.8 Hz, 2H), 1.67 – 1.43 (m, 6H), 1.31 – 1.20 (m, 10H), 0.93 – 0.83 (m, 3H).
HRMS: calcd for $[C_{14}H_{25}N]^+$ 210.22163; found 210.22148

Cooperative Effect of Rh/N-Xantphos and 2F on The Reductive Amination with Piperidine

The NMR of N-Xantphos ligand was recorded in toluene-d8/methanol d4 solvent mixture in NMR tubes prepared and sealed under nitrogen with the following additives: dicyclohexylamine (100 equivalents) and **2F** (2.5 equivalents) and the peak positions are reported in **Supplementary Table 2**. Following **General Procedure 2**, 0.515 ml nonanal and 0.296 ml piperidine were dissolved in toluene/methanol solution (4 to 1 volumetric ratio, 3 ml total, or 1 M nonanal) in an 8-ml glass vial. The reaction was performed at 115°C for 60 min residence time after reaching thermal equilibrium. The cold pressure was 24 barg and the initial H_2/CO ratio was set at 3.5. 50 μl aliquot was taken for analysis by GC-MS following the reaction completion (**Supplementary Table 3**, entry 1). In a second experiment, 9.24 mg of the co-catalyst **2F** were weighed in an 8-ml glass vial and then the reactants and solvent were added before starting the experiment (**Supplementary Table 3**, entry 2). In a third experiment, the reactants were dissolved in 1.5 ml toluene/methanol stock solution containing 0.77 mg of Rh catalyst and 6.6 mg of the ligand, then diluted with extra solvent to 3 ml total volume before starting the experiment (**Supplementary Table 3**, entry 3).

Supplementary Table 2. *^{1}H and ^{31}P NMR Studies of N-Xantphos in methanol/toluene deuterated solvent with acid and base additives.*

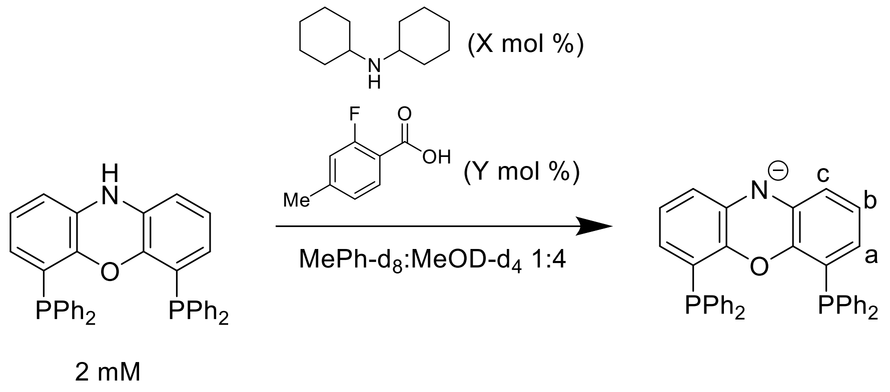

Entry	X mol (%)	Y mol (%)	pH	H^a	H^b	H^c	*P*Ph2	F*
1	0	0	5	5.82	6.32	6.42	-19.12	--
2	0	400	4	5.81	6.31	6.42	-19.11	-112.01
3	100,00	0	10	5.86	6.37	6.47	-19.02	--
4	100,00	250	10	5.85	6.36	6.46	-19.02	-115.94

* *^{19}F NMR shift for free **2F** is - 111.96 ppm*

Supplementary Table 3. *Effect of the acid-base additives on condensation/hydrogenation in methanol/toluene.*

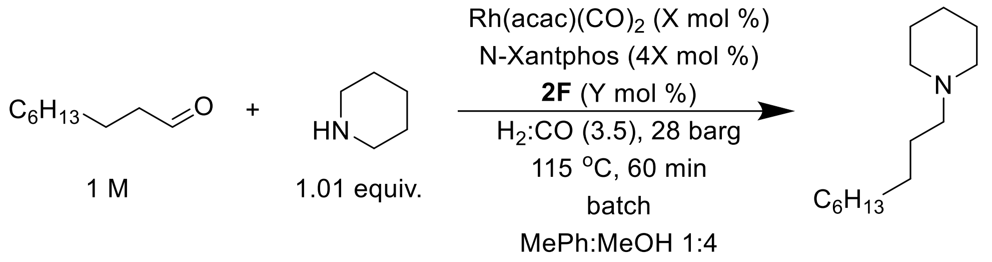

Entry	X mol %	Y mol %	Aldehyde Yield (%)	Amine Yield (%)	Enamine Yield (%)	Aldol Yield (%) [a]
1	0	0	24.3	0	64.1	11.6
2	0	2	19.9	0	57.8	22.3
3	0.1	2	0.0	92.2	3.7	4.1

[a] *calculated as aldehyde consumption% - (amine +enamine) yield %*

[b] *calculated as dicyclohexylamine consumption%-(amine +enamine) yield %*

Supplementary Table 4. *The cooperative effect of Rh/N-Xantphos and **2F** on condensation/reductive amination with dicyclohexylamine.*

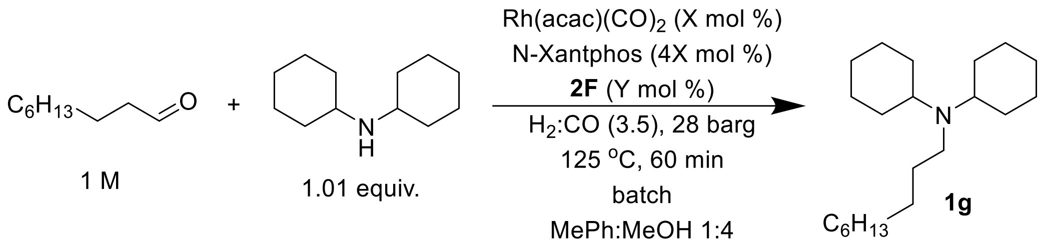

Entry	X mol %	Y mol %	Aldehyde Yield (%)	Amine Yield (%)	Enamine Yield (%)	Aldol Yield (%) [a]
1	0	0	89.8	0	2.9 [b]	7.3
2	0	10	77.7	0	3.0 [b]	19.3
3	0.1	0	72.9	20.2	2.1[b]	4.8
4	0.1	10	51.4	39.7	1.6 [b]	7.3

[a] *calculated as aldehyde consumption % - (amine+enamine) yield %*

[b] *calculated as dicyclohexylamine consumption % - (amine +enamine) yield %*

H/D Scrambling Experiment on the Reductive Amination with and without 2F

Following **General Procedure 2**, 0.515 ml nonanal and 0.296 ml piperidine were dissolved in toluene/methanol solution (4 to 1 volumetric ratio, 3 ml total or 1 M nonanal), containing 0.77 mg of Rh catalyst and 6.6 mg of the ligand in an 8-ml glass vial. The reaction was performed at 115°C for 30 min residence time after reaching thermal equilibrium. The cold pressure was 9.5 barg and the initial H_2/CO ratio was set at 0.875. 50 µl aliquot was taken for analysis by GC-MS following the reaction completion (**Supplementary Table 5**, entry 1). In a second experiment, the autoclave was pressurized with D_2 gas instead of H_2 (**Supplementary Table 5**, entry 2). In a third experiment, 9.24 mg of the co-catalyst **2F** were weighed in an 8-ml glass vial and then the reactants and solvent were added before starting the experiment (**Supplementary Table 5**, entry 3). In a fourth experiment, 9.24 mg of the co-catalyst **2F** were weighed in an 8-ml glass vial and then the reactants and solvent were added before starting the experiment with D_2 gas instead of H_2 (**Supplementary Table 5**, entry 4).

Supplementary Table 5. *H/D scrambling experiment on the reductive amination with and without **2F**.*

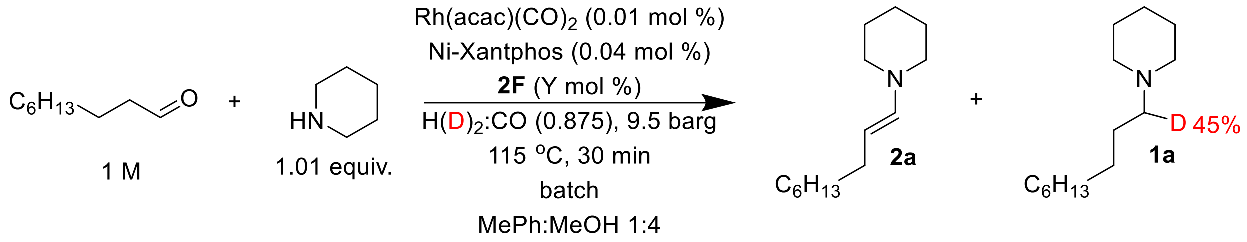

Entry	Y (mol %)	Gas	2g Yield (%)	1g Yield (%)	Hydrogenation (%) [a]	H/D (% Hydrogenation)
1	Zero	H_2	43.9	30.6	41.1	1.13
2		D_2	48.0	27.3	36.3	
3	2	H_2	32.4	47.1	59.2	1.03
4		D_2	32.5	44.1	57.6	

H/D Scrambling Experiment on the Hydroformylation of 1-octene under HAM Conditions

Following **General Procedure 2**, 0.47 ml of 1-octene was dissolved in toluene/methanol solution (4 to 1 volumetric ratio, 3 ml total, or 1 M 1-octene), containing 0.77 mg of Rh catalyst and 6.6 mg of the ligand in an 8-ml glass vial. The reaction was performed at 115°C for 30 min residence time after reaching thermal equilibrium. The cold pressure was 9.5 barg and the initial H_2/CO ratio was set at 0.875. 50 μl aliquot was taken for analysis by GC-MS following the reaction completion (**Supplementary Table 6**, entry 1). In a second experiment, the autoclave was pressurized with D_2 gas instead of H_2 (**Supplementary Table 6**, entry 2). In a third experiment, 9.24 mg of the co-catalyst **2F** were weighed in an 8-ml glass vial and then the reactants and solvent were added before starting the experiment (**Supplementary Table 6**, entry 3).

Supplementary Table 6. *H/D scrambling experiment on the hydroformylation of 1-octene under HAM conditions.*

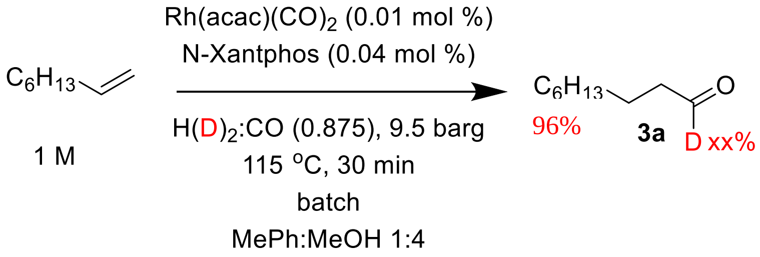

Entry	2F (mol %)		3a Yield (%)	H/D (% Hydroformylation)
1	Zero	H_2	11.9	**1.03**
2	Zero	D_2	11.6	
3	2	H2	11.3	

HAM at Variable Residence Time and Gas to Liquid ratio (Supplementary Fig. 2)

Following **General Procedure 1**, the solution for loading in the catalyst syringe was prepared by dissolving 5.16 mg (0.1 mol %) of dicarbonyl 2,4-pentanedionato rhodium(I) catalyst and 44.1 mg of N-Xantphos ligand in 2 ml toluene. The solution was diluted with 8 ml methanol solvent and 1 ml of this solution was diluted in 9 ml toluene/methanol solvent (4:1 volumetric ratio) to achieve the desired catalyst loading (0.01 mol %). An 8-ml stainless steel syringe was filled with the diluted catalyst solution. 1-octene (3.13 ml, 1 M) and piperidine (1.97 ml, 0.99 equiv.) were added to 61.6 mg of co-catalyst **2F** (2.0 mol %), diluted with toluene/methanol solvent to 10 ml total volume, and loaded into an 8-ml stainless steel syringe. The flow reactor pressure was set at 28 barg. The syringe pump flowrates as well as the H_2 and CO flow rates were varied to tune the residence time in the flow reactor at constant gas to liquid volumetric ratio and H_2/CO ratio of 5 and 3.5, respectively. The flow reactor temperature was set at 115°C. The reaction was run for 40 min before collecting samples for another 40 min. The collected crude mixture was analyzed by GC-MS. In a separate experiment, the liquid and gas flow rates were varied to vary the gas to liquid ratio at constant residence time and H_2/CO ratio of 25 min and 3.5, respectively.

HAM TOF Calculations

To measure the turnover frequency (TOF) of both hydroformylation and enamine hydrogenation reaction that can be achieved in flow under typical HAM conditions, the catalyst loading was reduced from 0.1 to 0.01 mol% and the 1-octene reaction with piperidine was performed under variable residence times. The ligand to Rh ratio was set at 4 and the co-catalyst **2F** loading was set at 2 mol %. The hydroformylation TOF is calculated as the number of moles (aldehyde + enamine + amine)/mole Rh/h and the HAM TOF is calculated as the of moles of amine/mole Rh/h at 115°C and gas to liquid volumetric ratio of 5. The initial hydroformylation TOF was 9,000 mol/mol Rh/h, while the HAM TOF under optimized reaction conditions was 4,000 mol/mol Rh/h (**Supplementary Fig. 2(A)**). When the gas to liquid volumetric ratio is 10, the HAM TOF approaches 10,000 mol amine/mol Rh/h (**Supplementary Fig. 2(B)**).

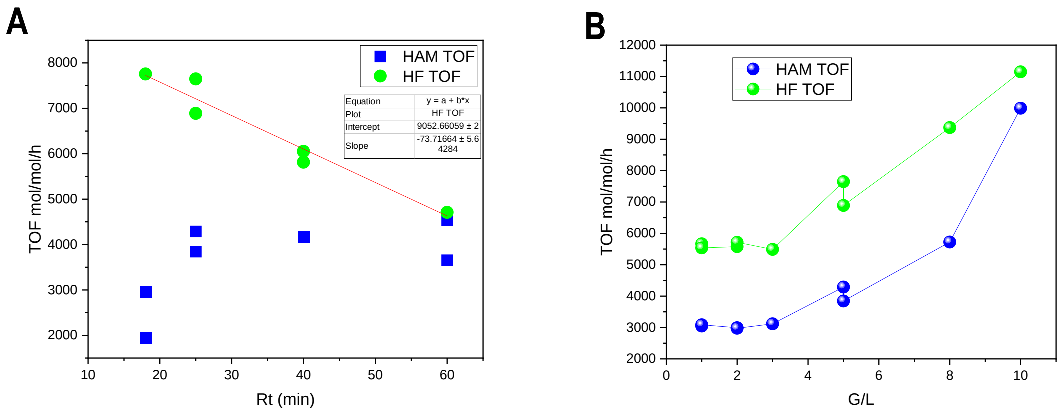

Supplementary Fig. 2. *TOF of in-flow HAM of 1-octene with piperidine in the presence of Rh/N-Xantphos and co-catalyst **2F** at 115°C and 28 barg (total pressure). Inlet H_2/CO ratio was set at 3.5 and solvent composition was 4:1 toluene /methanol (volumetric ratio). The catalyst loading was 0.01 mol% and the N-Xantphos to Rh ratio was 4. **2F** loading was 2 mol%. (a) Effect of residence time at gas:liquid volumetric ratio of 5. (b) Effect of gas:liquid volumetric ratio at a residence time of 25 min.*

Batch Reactor

A picture of the pressurized autoclave with a glass vial insert (Buchiglass Tinyclave) is shown in **Supplementary Fig. 3**.

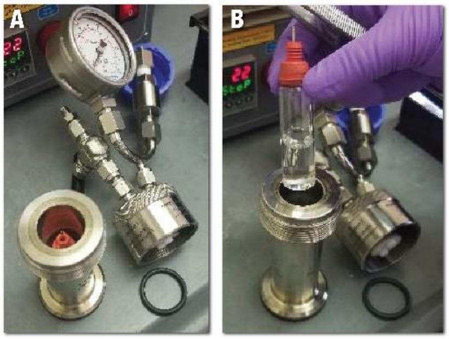

Supplementary Fig. 3. *(A) A picture of the pressurized autoclave used for batch reactions with (B) an 8-ml glass vial insert.*

NMR Spectra:

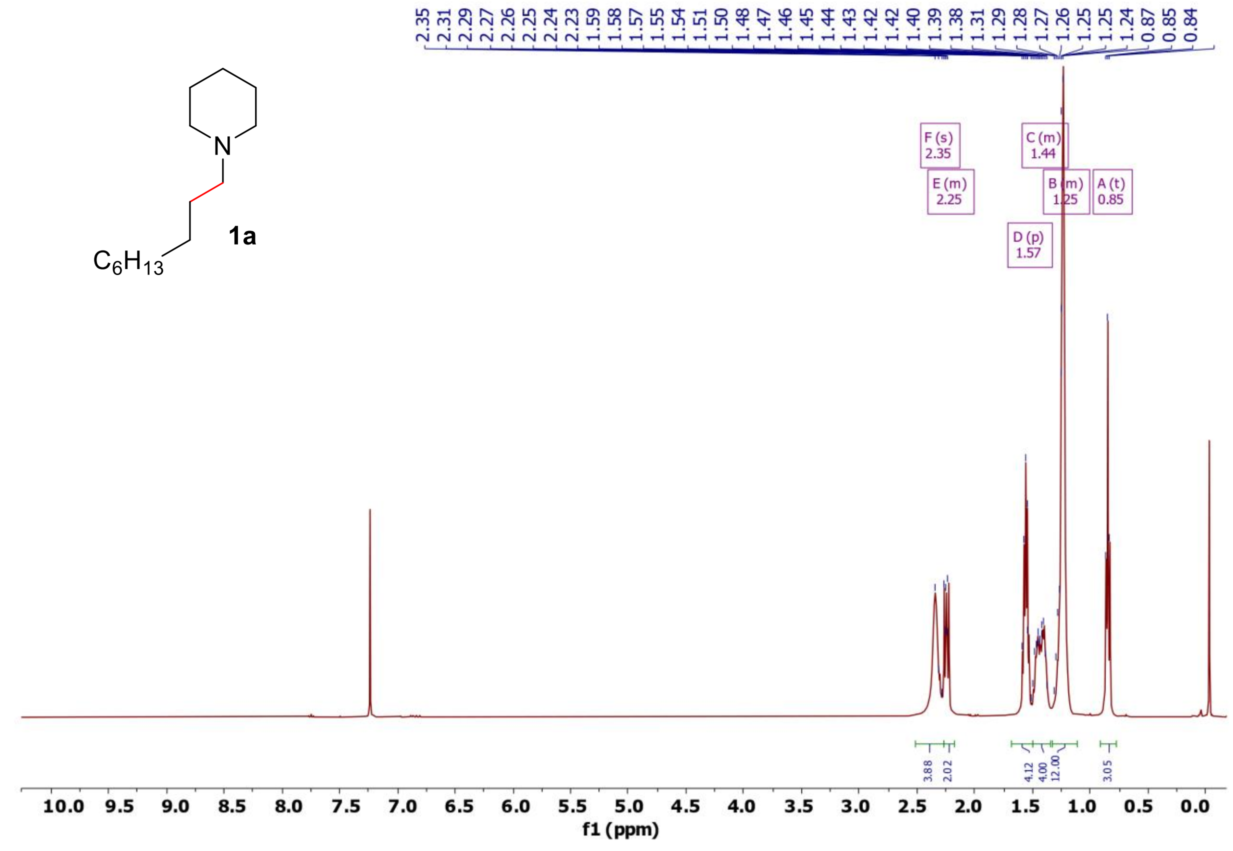

Supplementary Fig. 4. ^{1}H NMR spectra of compound **1a**

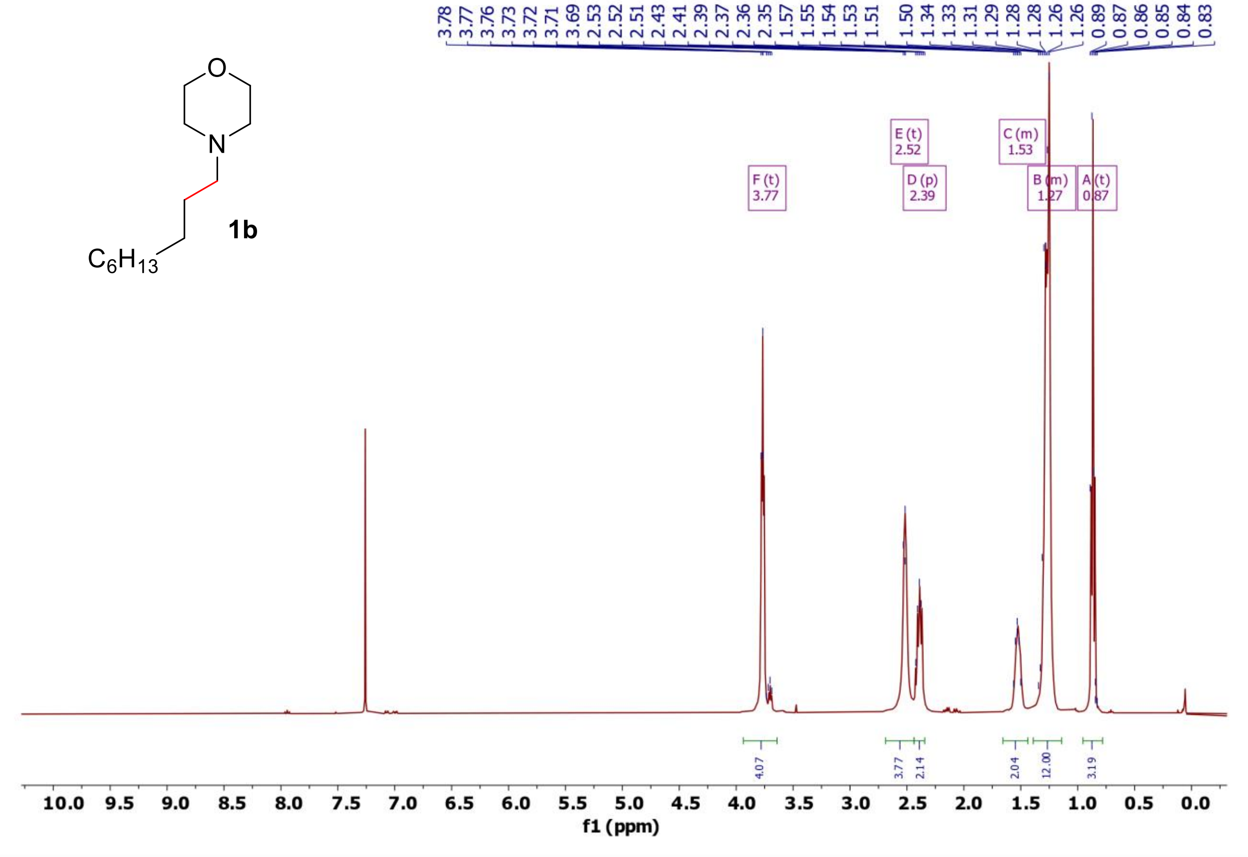

Supplementary Fig. 5. ^{1}H NMR spectra of compound **1b**

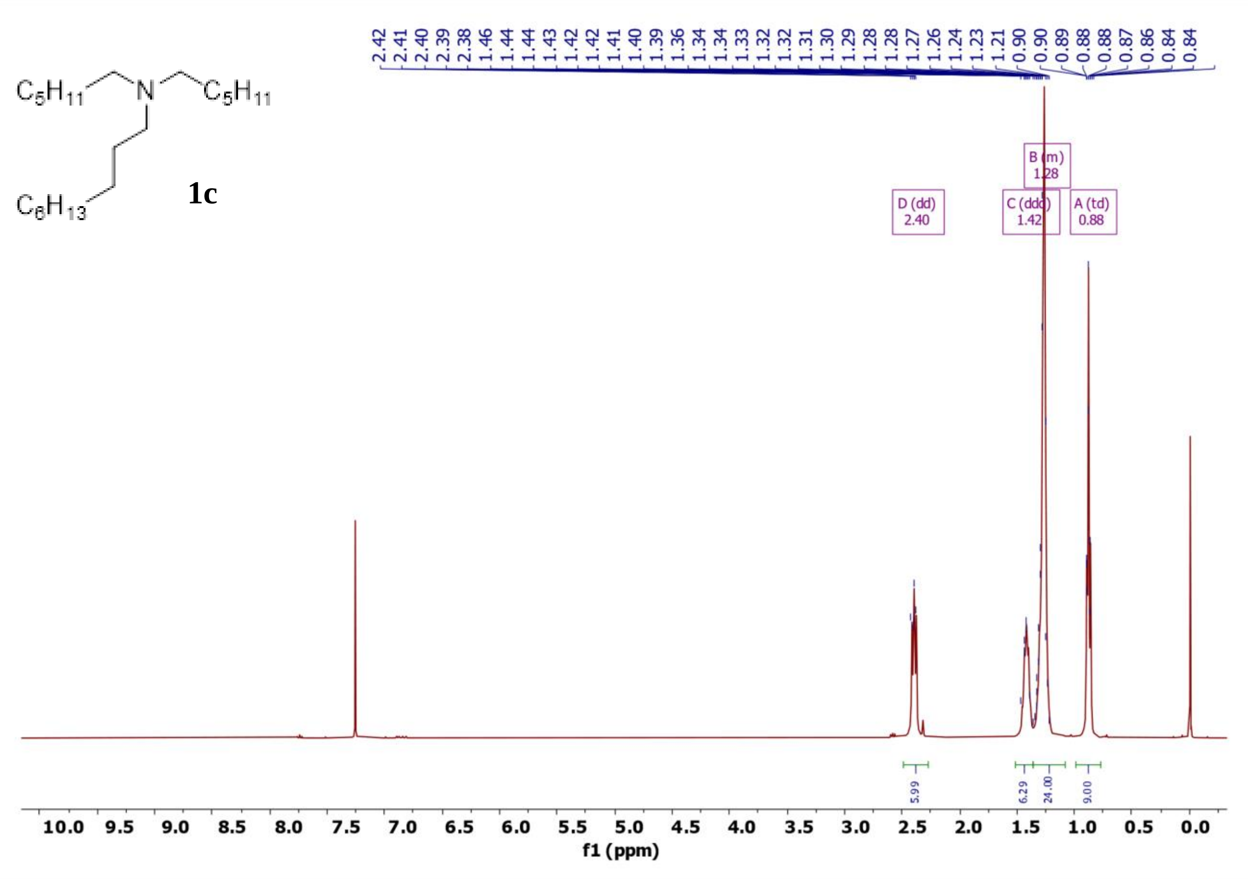

Supplementary Fig. 6. ^{1}H NMR spectra of compound **1c**

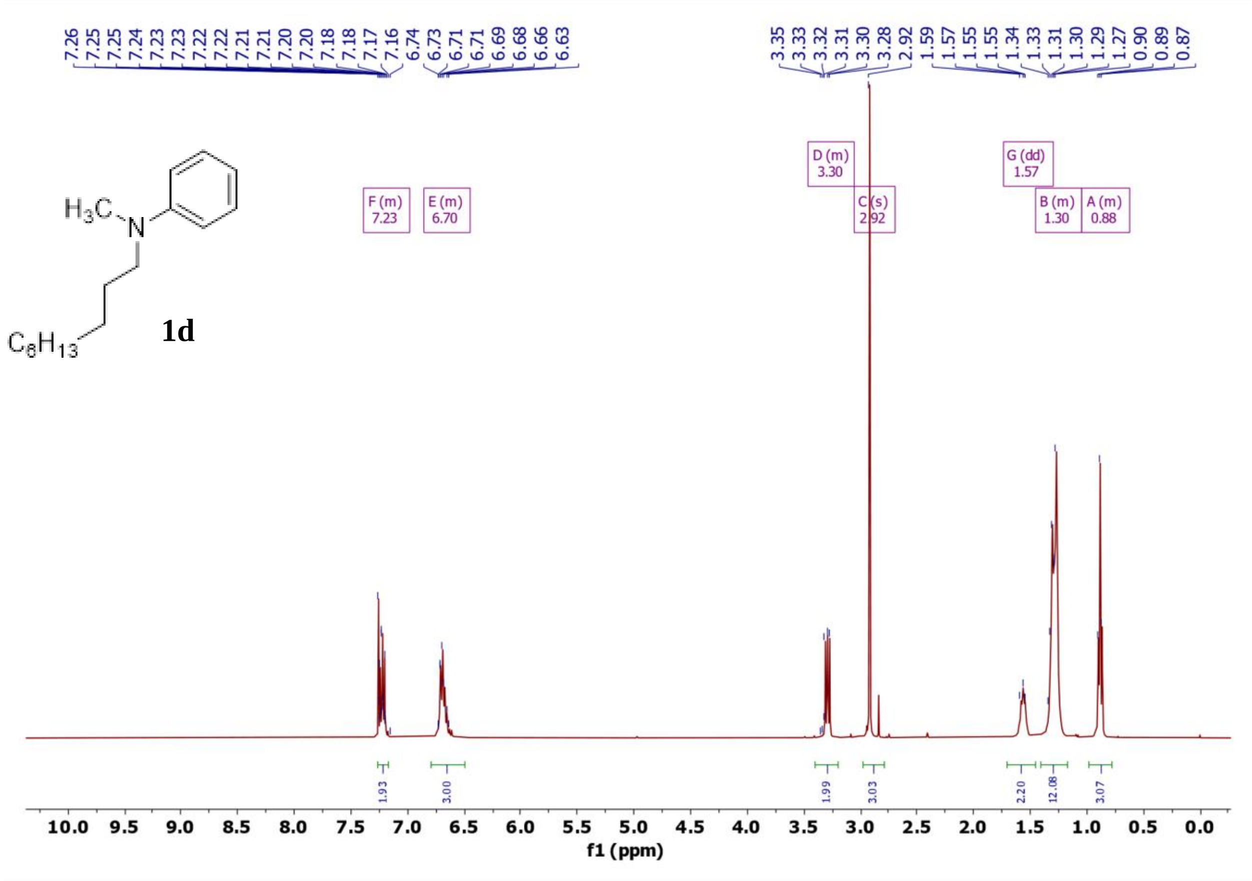

Supplementary Fig. 7. ^{1}H NMR spectra of compound **1d**

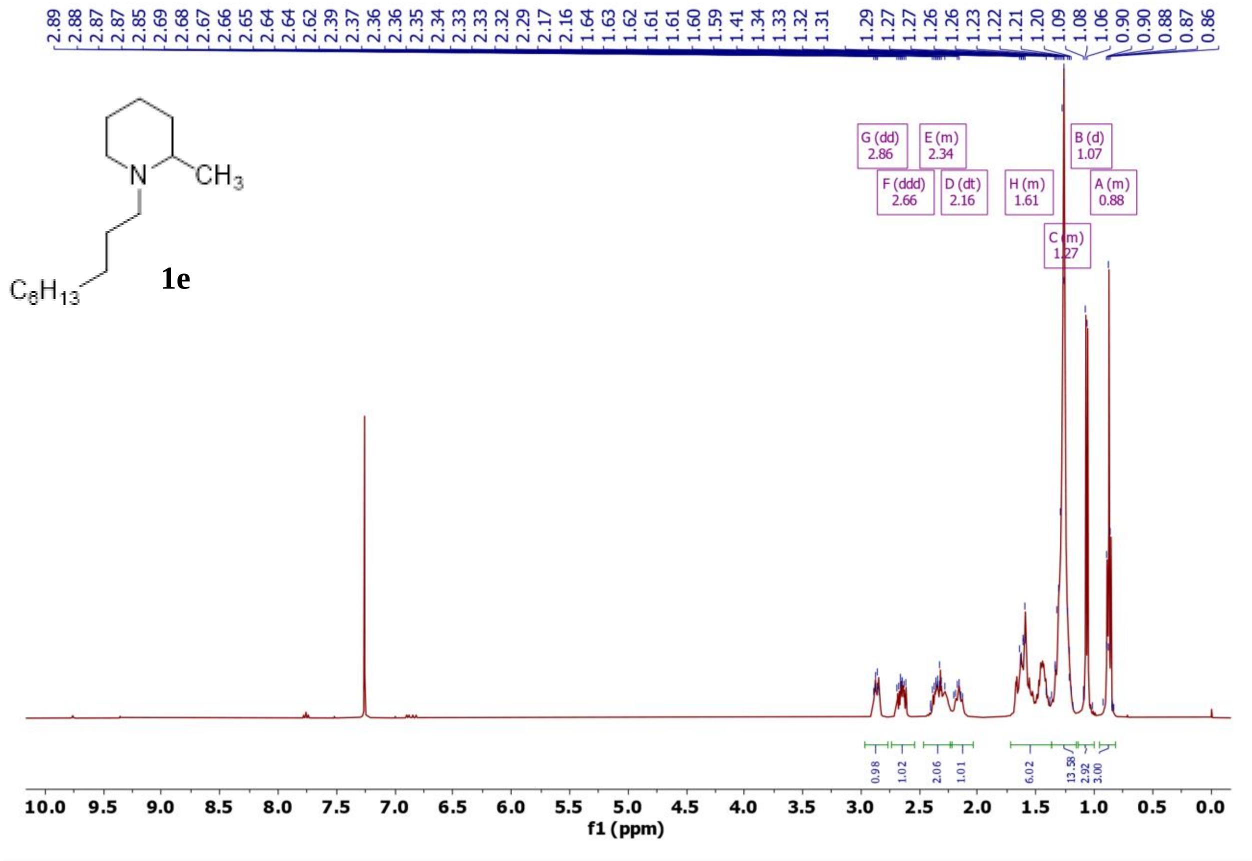

Supplementary Fig. 8. ^{1}H NMR spectra of compound **1e**

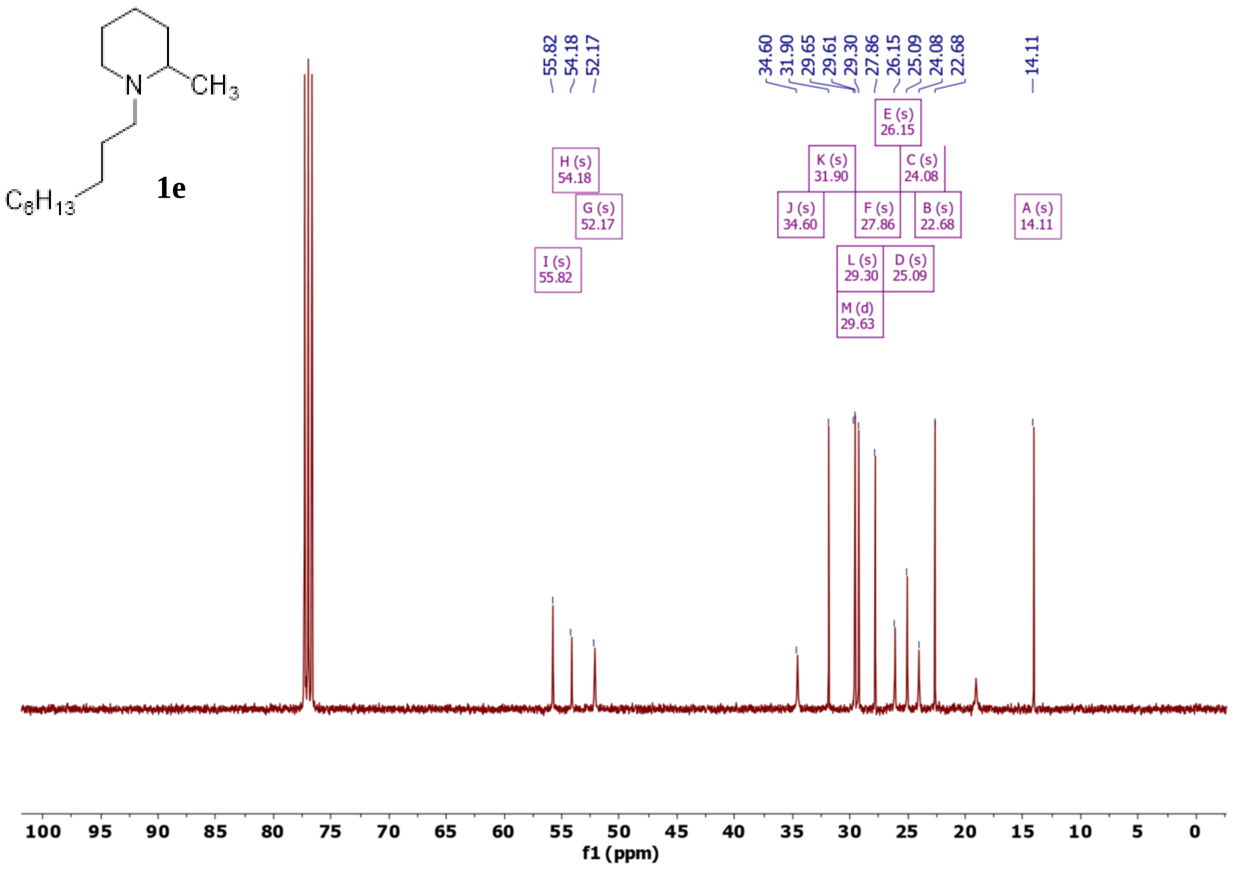

Supplementary Fig. 9. ^{1}H NMR spectra of compound **1e**

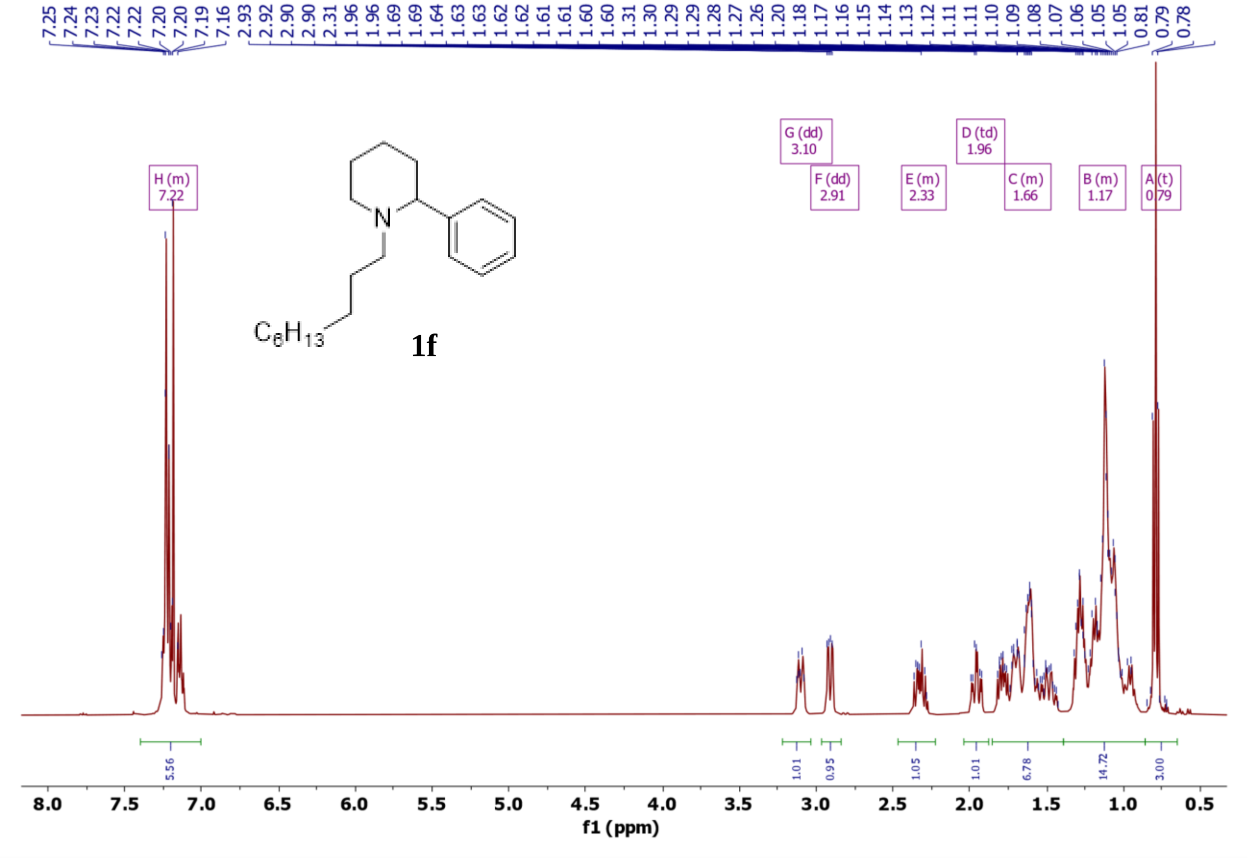

Supplementary Fig. 10. ^{1}H NMR spectra of compound **1f**

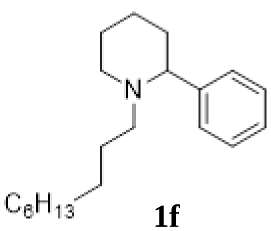

1f

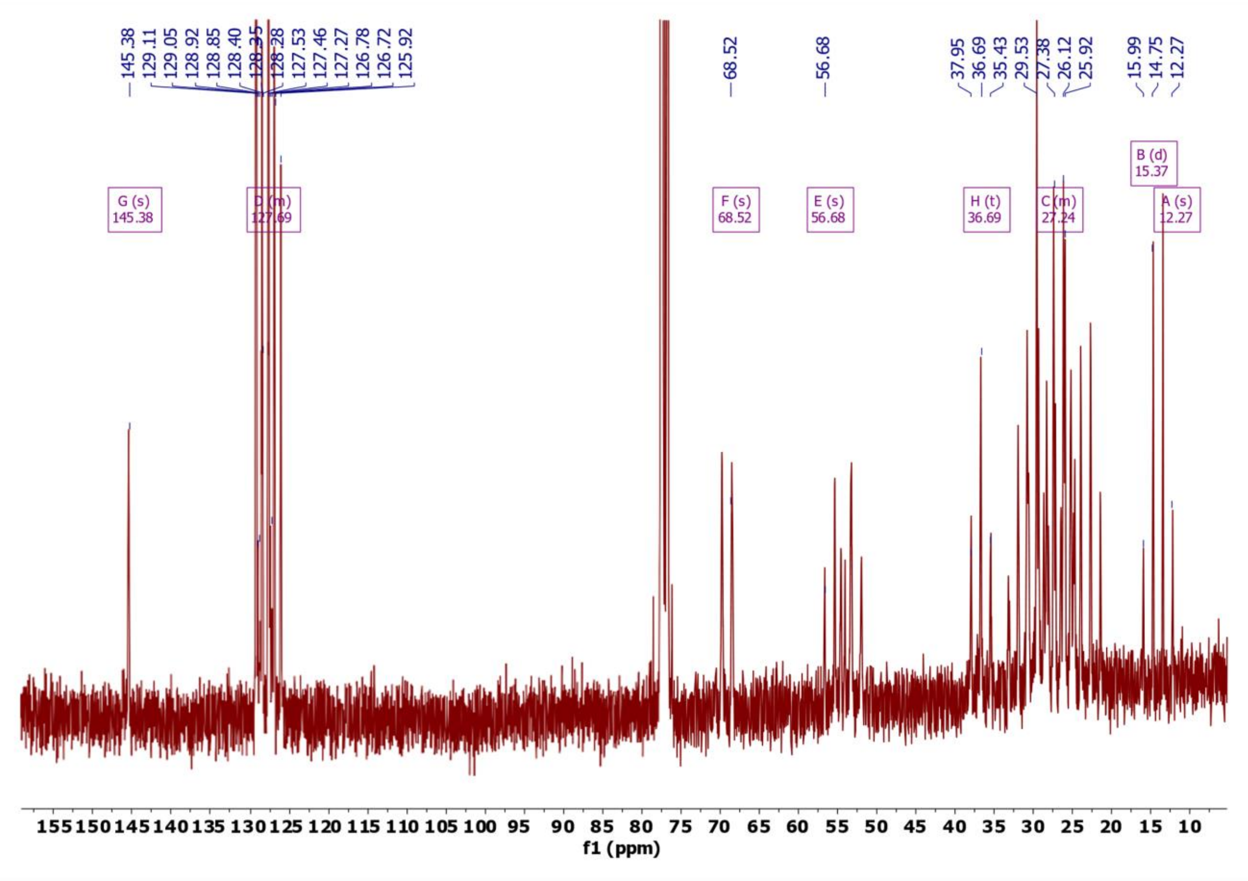

Supplementary Fig. 11. ^{1}H NMR spectra of compound **1f**

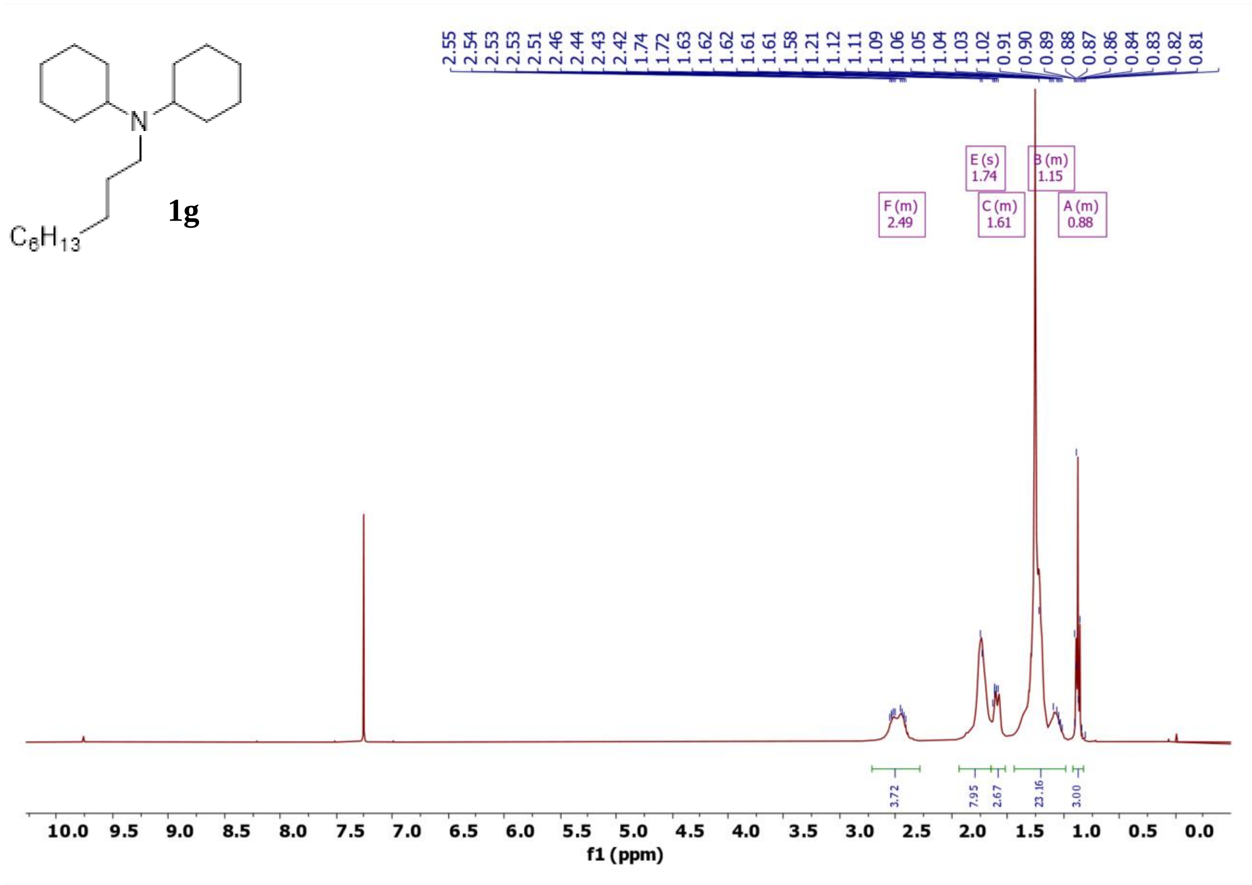

Supplementary Fig. 12. ^{1}H NMR spectra of compound **1g**

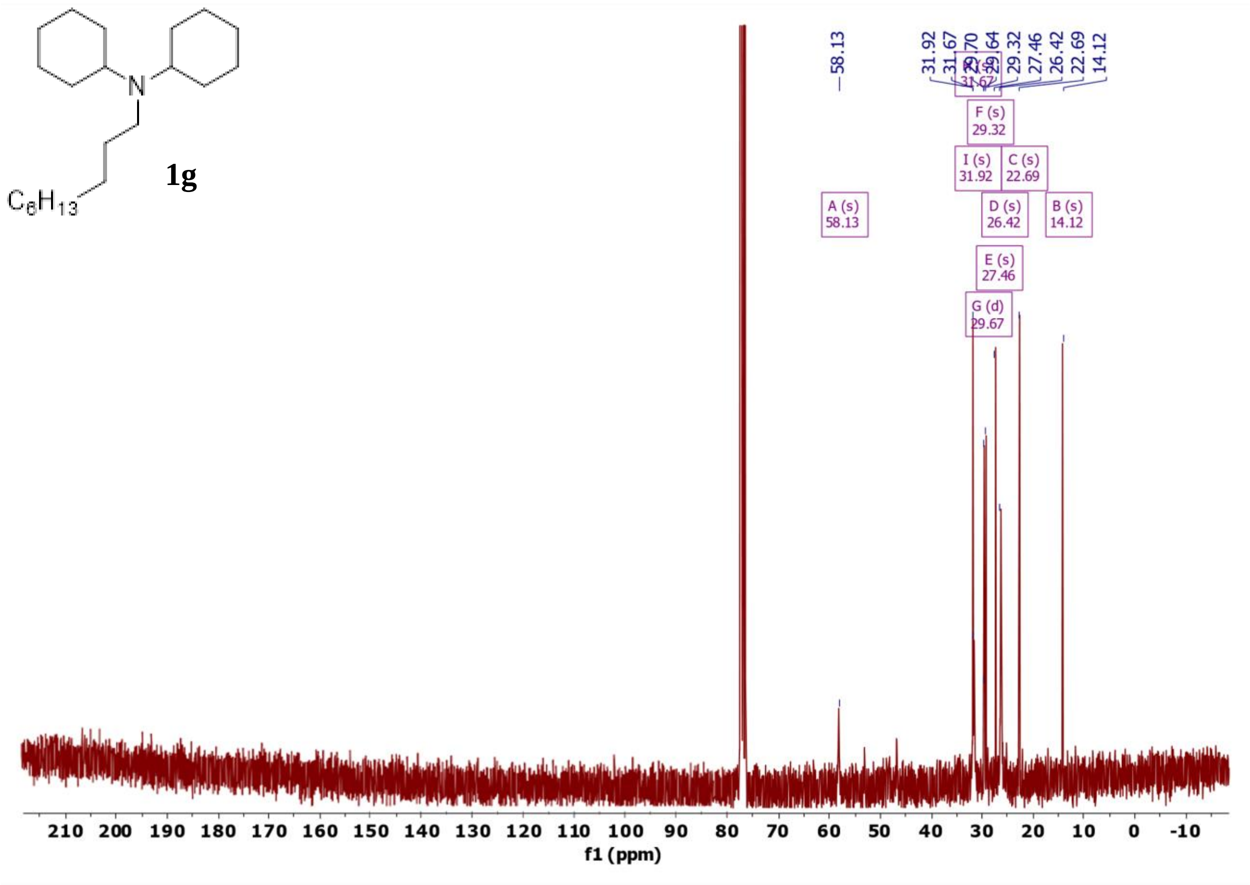

Supplementary Fig. 13. ^{13}C NMR spectra of compound **1g**

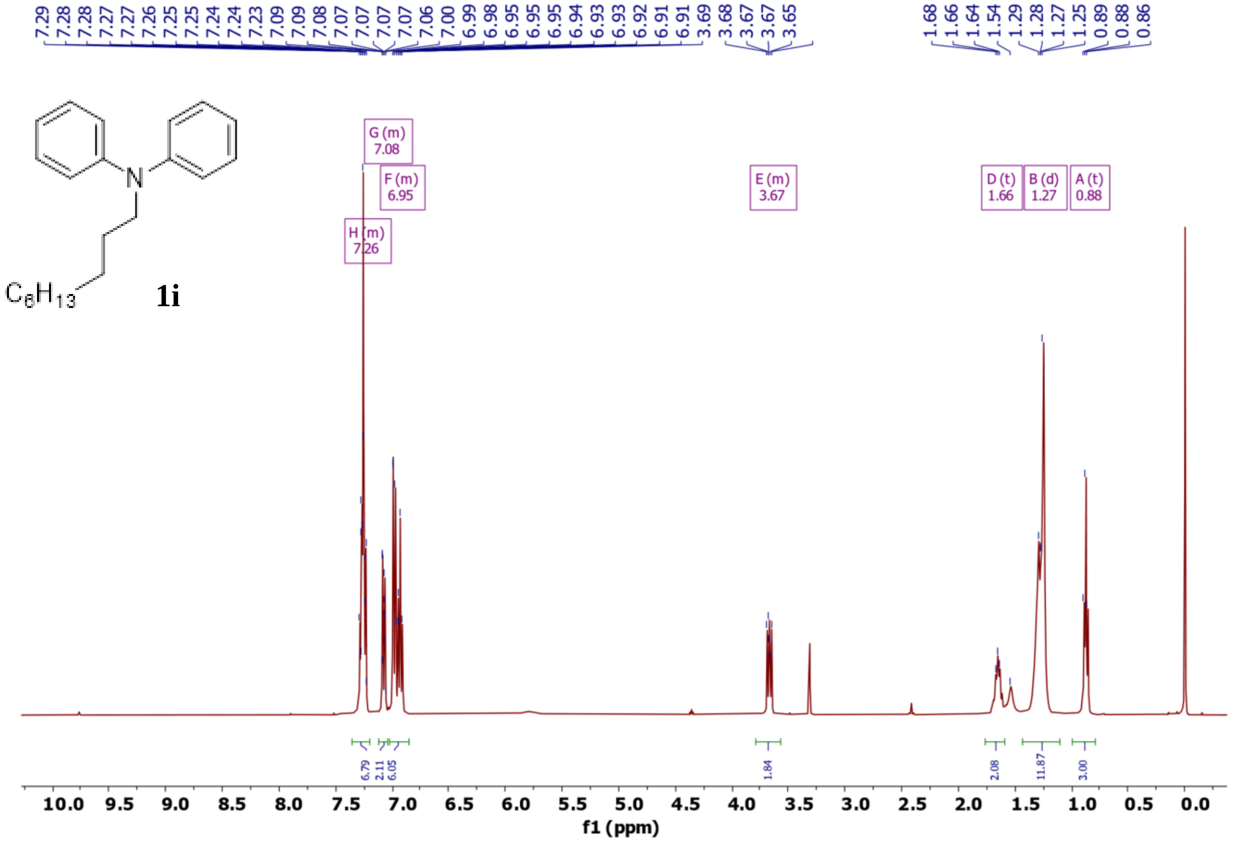

Supplementary Fig. 14. ^{1}H NMR spectra of compound **1i**

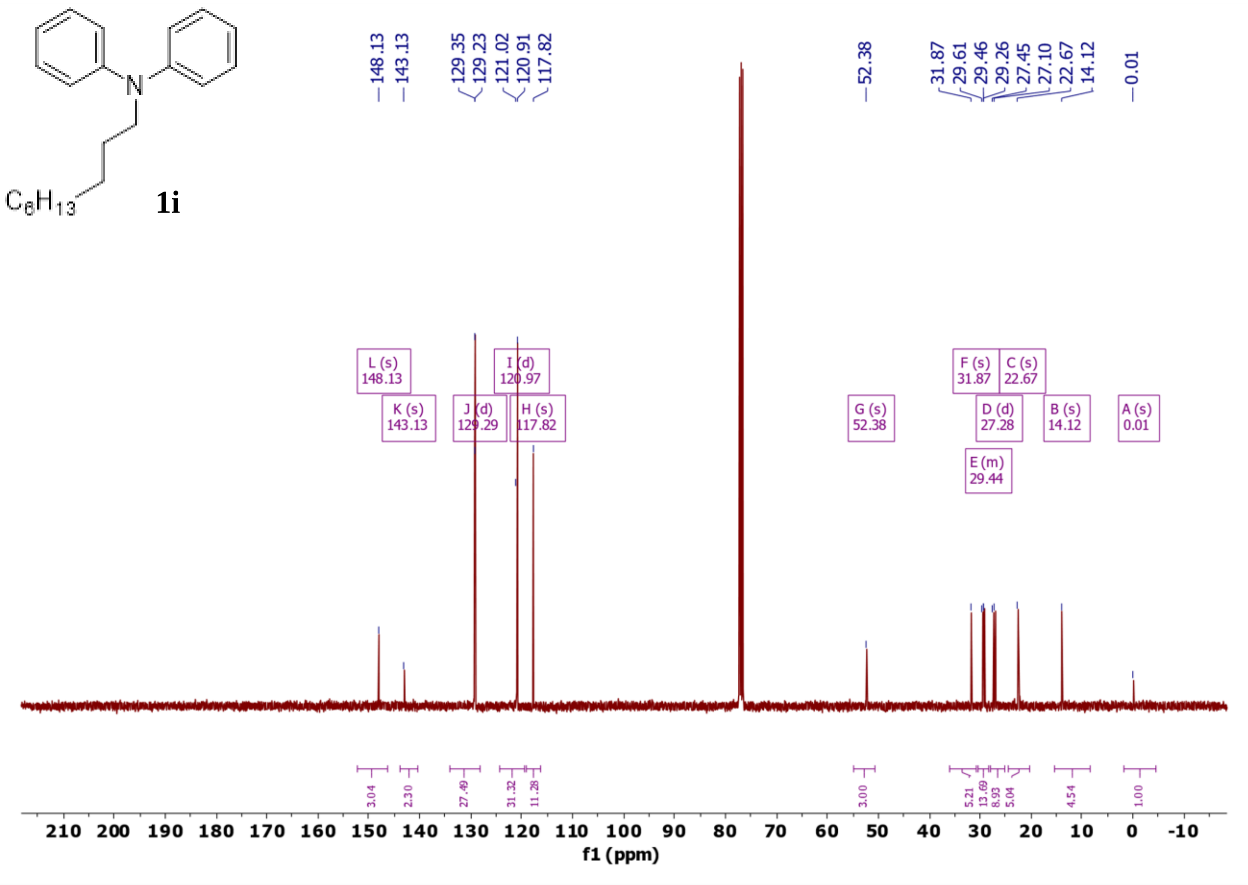

Supplementary Fig. 15. ^{1}H NMR spectra of compound **1i**

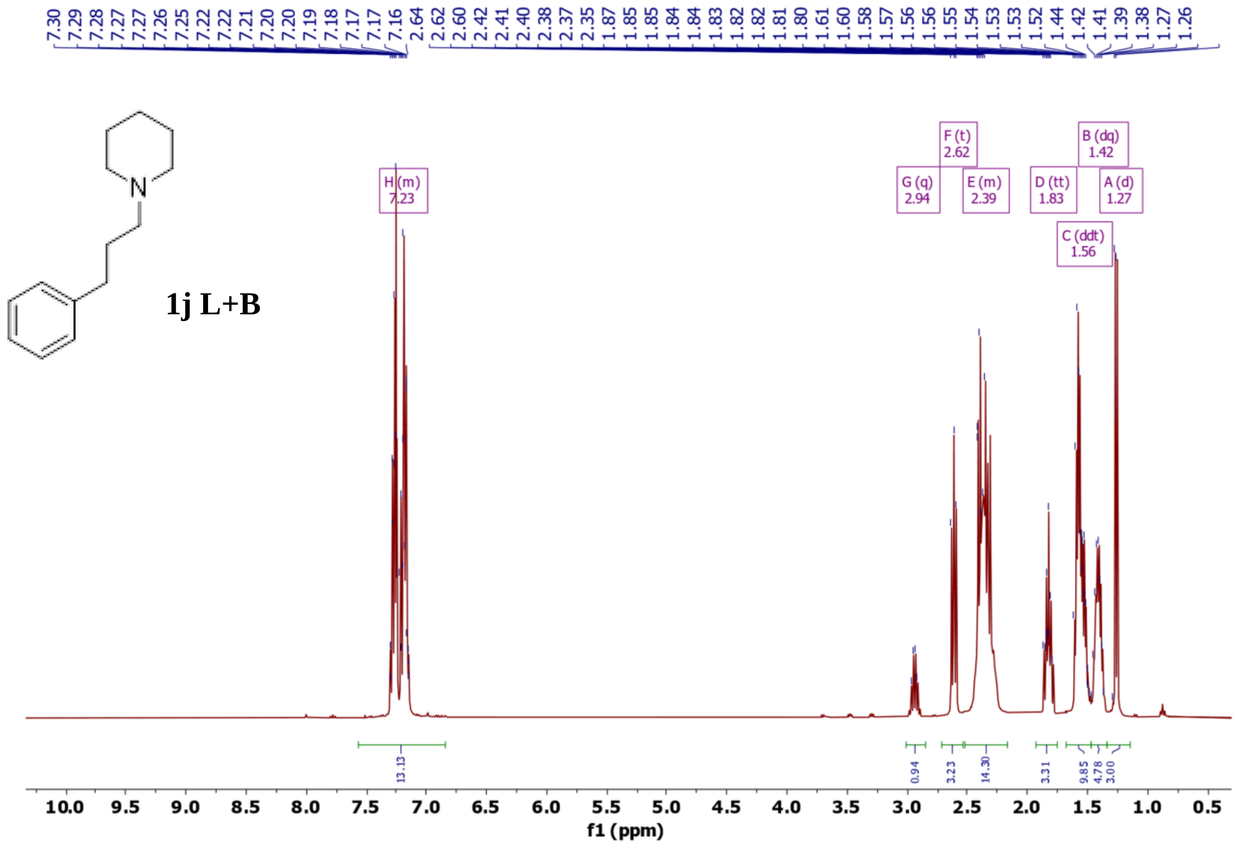

Supplementary Fig. 16. ^{1}H NMR spectra of compound **1j**

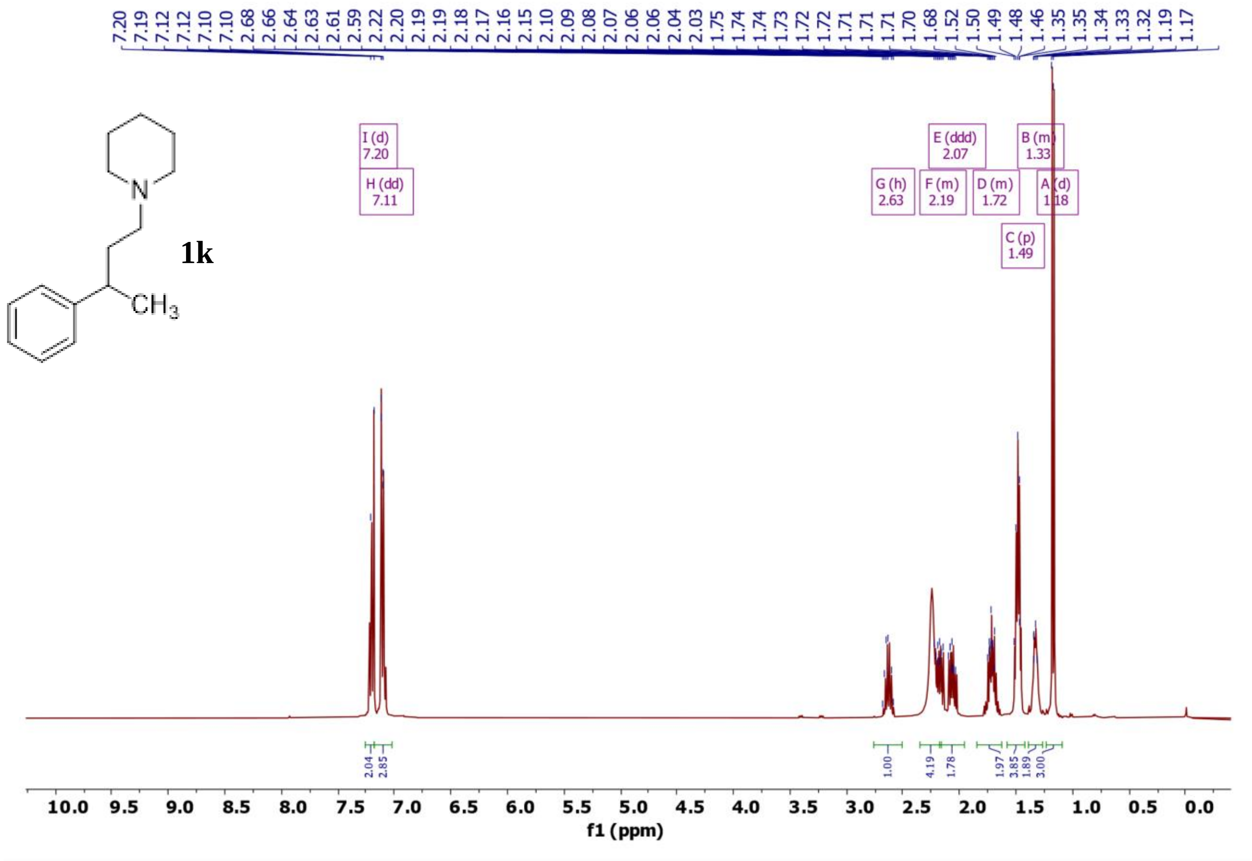

Supplementary Fig. 17. ^{1}H NMR spectra of compound **1k**

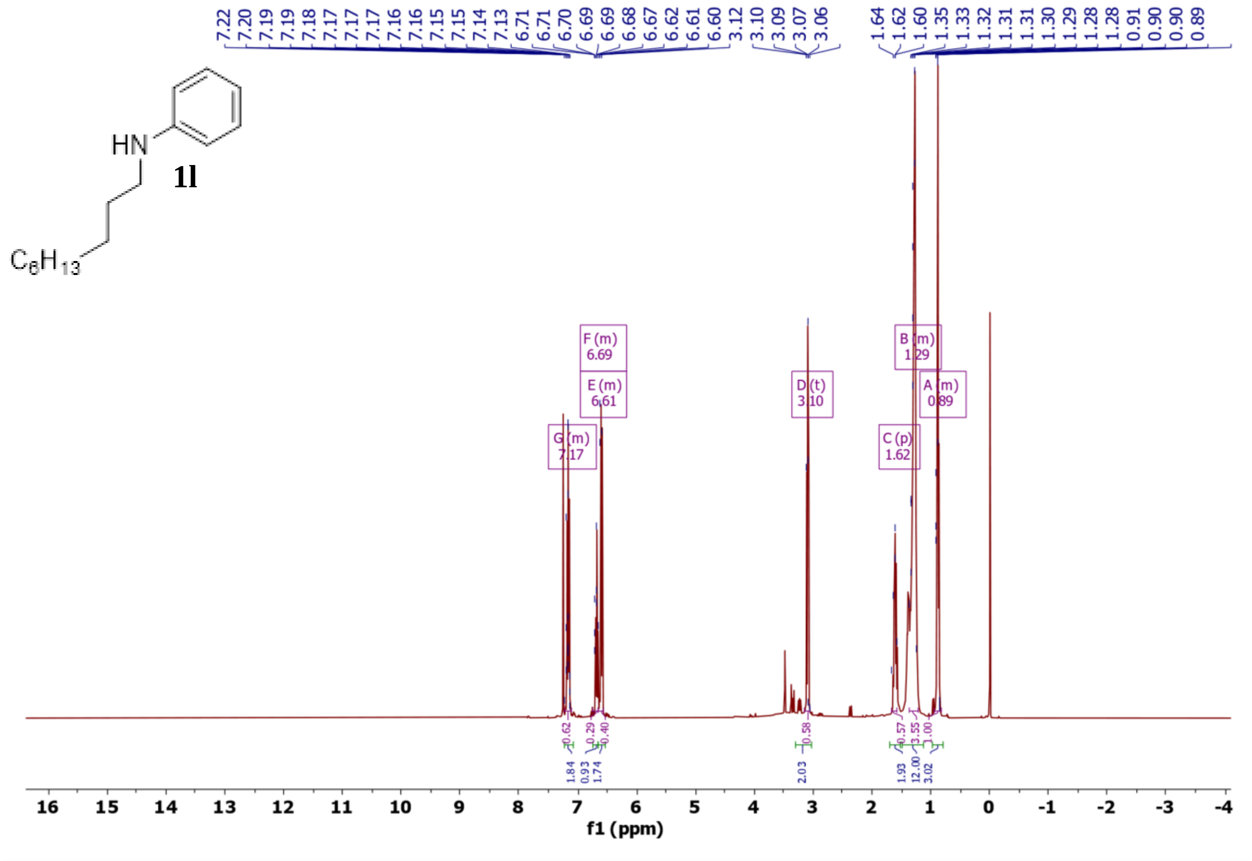

Supplementary Fig. 18. ^{1}H NMR spectra of compound **1l**

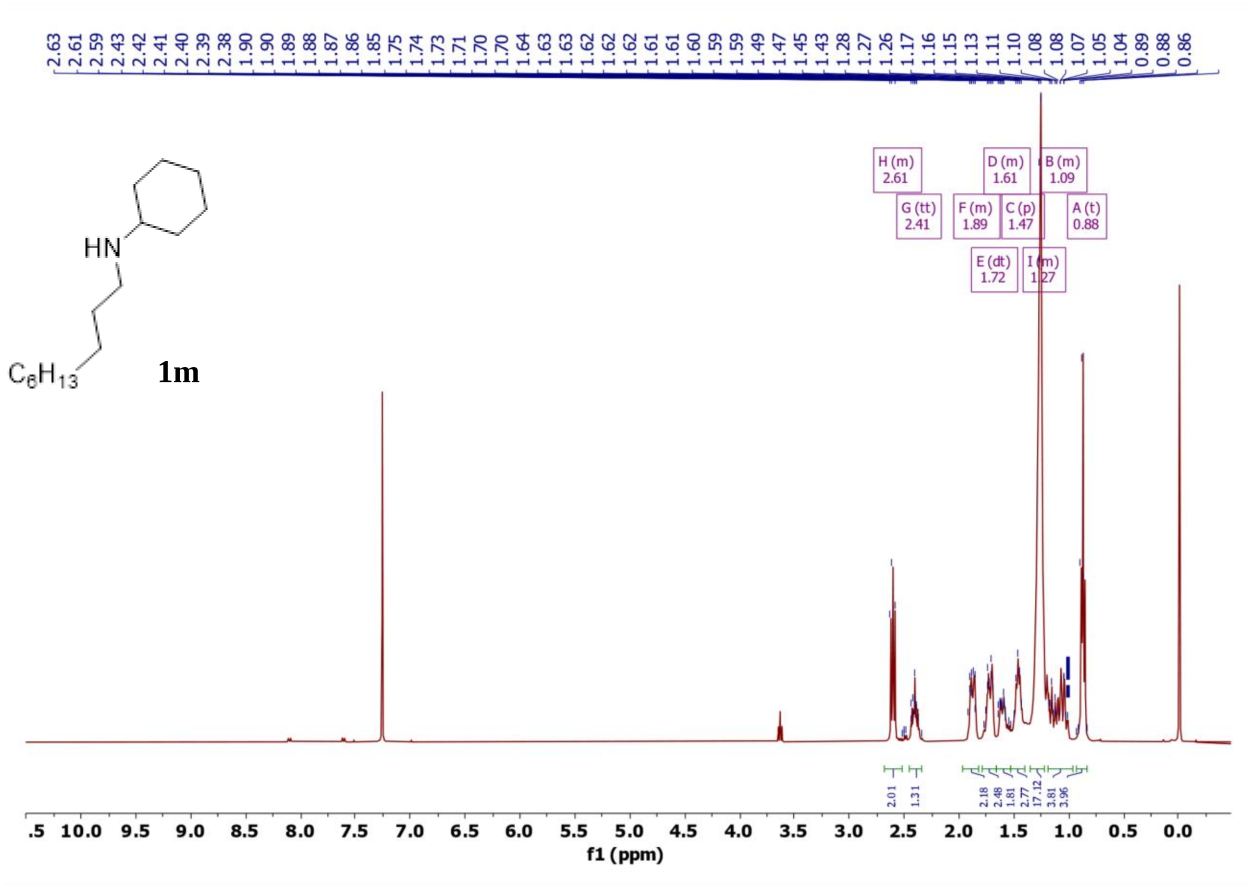

Supplementary Fig. 19. ^{1}H NMR spectra of compound **1m**

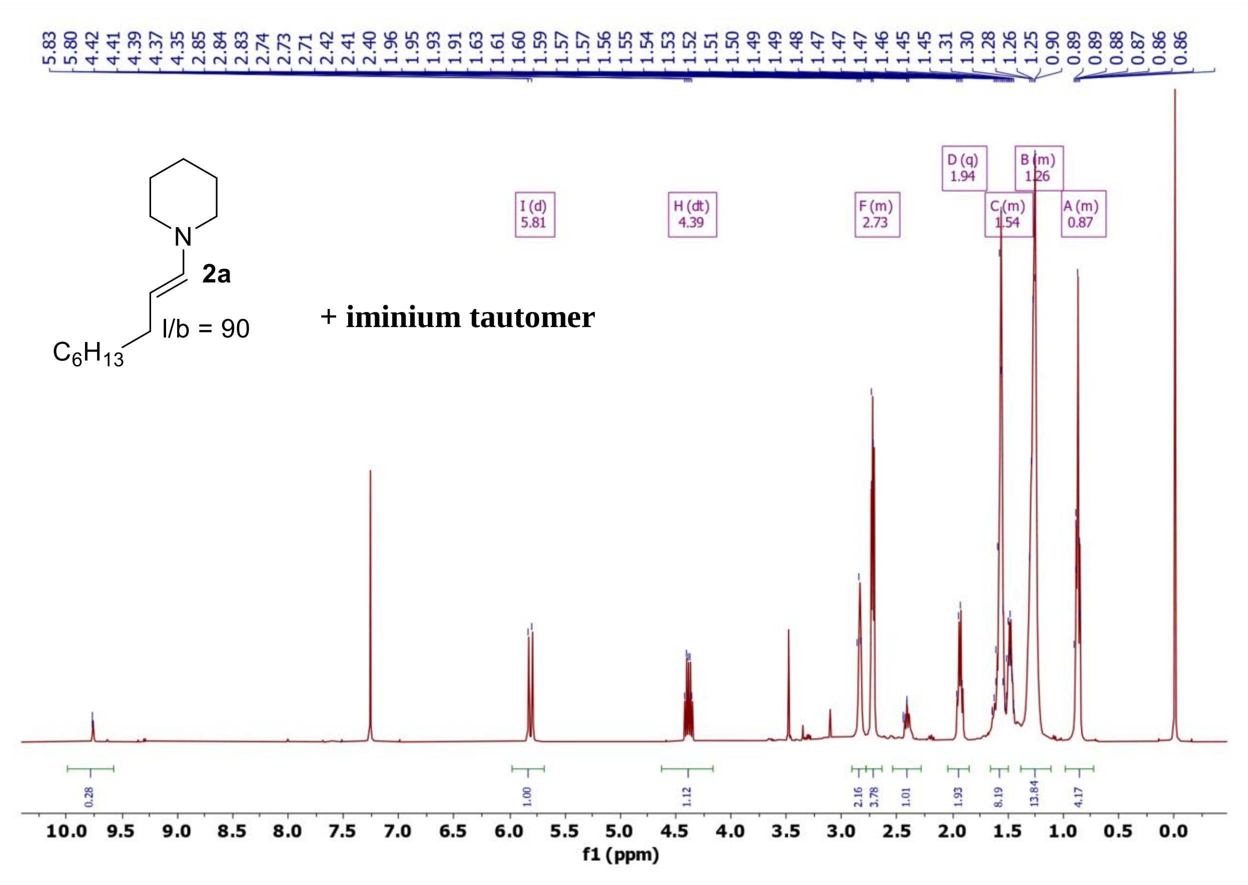

Supplementary Fig. 20. ^{1}H NMR spectra of compound **2a**

3. Supplementary References

1. L. Wu, L. Fleischer, R. Jackstell, M. Beller, Efficient and Regioselective Ruthenium-catalyzed Hydro-aminomethylation of Olefins. *J. Am. Chem. Soc.* **135**, 3989-3996 (2013).
2. H. Liu, D. Yang, D.-L. Wang, P. Wang, Y. Lu, V. o. T. Giang, Y. Liu, An efficient and recyclable ionic diphosphine-based Ir-catalyst for hydroaminomethylation of olefins with H_2O as the hydrogen source. *Chem. Commun.* **54**, 7979-7982 (2018).
3. M. Ahmed, A. M. Seayad, R. Jackstell, M. Beller, Amines Made Easily: A Highly Selective Hydroaminomethylation of Olefins. *J. Am. Chem. Soc.* **125**, 10311-10318 (2003).
